# A Shift to Organismal Stress Resistance in Programmed Cell Death Mutants

**DOI:** 10.1371/journal.pgen.1003714

**Published:** 2013-09-19

**Authors:** Meredith E. Judy, Ayumi Nakamura, Anne Huang, Harli Grant, Helen McCurdy, Kurt F. Weiberth, Fuying Gao, Giovanni Coppola, Cynthia Kenyon, Aimee W. Kao

**Affiliations:** 1 Memory and Aging Center, Department of Neurology, University of California San Francisco, San Francisco, California, United States of America; 2 Department of Biochemistry, University of California San Francisco, San Francisco, California, United States of America; 3 Semel Institute for Neuroscience and Human Behavior, Departments of Psychiatry and Biobehavioral Sciences and Neurology, David Geffen School of Medicine, University of California Los Angeles, Los Angeles, California, United States of America; Stanford University Medical Center, United States of America

## Abstract

Animals have many ways of protecting themselves against stress; for example, they can induce animal-wide, stress-protective pathways and they can kill damaged cells via apoptosis. We have discovered an unexpected regulatory relationship between these two types of stress responses. We find that *C. elegans* mutations blocking the normal course of programmed cell death and clearance confer animal-wide resistance to a specific set of environmental stressors; namely, ER, heat and osmotic stress. Remarkably, this pattern of stress resistance is induced by mutations that affect cell death in different ways, including *ced-3* (cell death defective) mutations, which block programmed cell death, *ced-1* and *ced-2* mutations, which prevent the engulfment of dying cells, and progranulin (*pgrn-1*) mutations, which accelerate the clearance of apoptotic cells. Stress resistance conferred by *ced* and *pgrn-1* mutations is not additive and these mutants share altered patterns of gene expression, suggesting that they may act within the same pathway to achieve stress resistance. Together, our findings demonstrate that programmed cell death effectors influence the degree to which *C. elegans* tolerates environmental stress. While the mechanism is not entirely clear, it is intriguing that animals lacking the ability to efficiently and correctly remove dying cells should switch to a more global animal-wide system of stress resistance.

## Author Summary

As an animal interacts with its environment, it invariably encounters stressful conditions such as extreme temperatures, drought, UV exposure and harmful xenobiotics. Since the ability to respond appropriately to stressful stimuli is paramount to survival, organisms have developed sophisticated stress response programs. Some stressful conditions cause damaged cells to commit suicide (undergo apoptosis), whereas others cause the entire organism to develop mechanisms to resist environmental stress. Studying the small roundworm *C. elegans*, we find that these two responses are somehow linked: perturbing the mechanisms that allow cells to undergo apoptosis changes the whole animal's response to environmental stress. In fact, perturbing the apoptosis machinery in any way—through mutations that prevent apoptosis altogether, or through mutations that either slow or accelerate the clearance of dying cells—causes the animal to become more stress resistant. Together our findings raise the possibility that the animal may have a way of detecting defects in the normal programmed cell death pathway, and that in response it induces a new program that protects itself from a harsh environment.

## Introduction

In nature, animals are constantly exposed to changing environmental conditions. In order to survive, organisms must weather normal and oftentimes extreme variations in temperature, water availability, salt levels, xenobiotics and other environmental factors. To cope, animals have developed mechanisms for stress protection. At the cellular level, DNA damage and other forms of stress can induce apoptosis to remove damaged cells; at the organismal level, stressful conditions can sometimes induce responses that make the entire animal more stress resistant [1], [2].

The nematode *C. elegans* has evolved several stress-protective responses. These include aversive behaviors, such as avoidance of noxious stimuli, and the activation of alternative developmental programs, such as entry into the dauer state of diapause [3], [4]. The animal can also induce environmental stress resistance by turning on gene transcription to manage the stressor using, for example, the heat-shock transcription factor HSF-1 to combat heat stress [5], SKN-1/Nrf2 to combat xenobiotic stress [6] and the hypoxia-inducible factor HIF-1 to combat hypoxia [7].

In addition to coordinated stress responses at the organismal level, *C. elegans*, like other organisms, can protect itself against stress at the cellular level. For example, in *C. elegans*, germ cells undergo apoptosis in response to DNA damage from ionizing radiation [8], [9]. In general, these types of single-cell, live-or-die decisions may be made to sacrifice a part for the betterment of the whole. How these decisions are made and the mechanistic and molecular relationship, if any, between animal-wide stress responses and programmed cell death are, however, poorly understood.

As a fundamental process by which organisms remove unnecessary, abnormal or damaged cells, programmed cell death involves both cell killing via apoptosis and cell corpse removal via phagocytosis and degradation [10]. Although once considered a disinterested second party that simply removes the dead cell, the engulfing cell is now known to be an active participant in the cell death program. For example, in weak *C. elegans* caspase mutants, in which decisions about whether to complete the cell death program are made stochastically, a second mutation in an engulfment gene further reduces cell death [11], [12]. Likewise, in mammals, mutations affecting either the dying or engulfing cell can disrupt tissue homeostasis and produce developmental disorders, autoimmune disease, cancer and neurodegeneration [13].

Genes responsible for carrying out apoptosis and apoptotic-cell engulfment were first described in *C. elegans* (Figure S1) [14], [15]. In the apoptotic cell, the canonical programmed cell death pathway involves the Apaf-1 like protein CED-4, which is inhibited by the BCL-2-like protein, CED-9 [16]–[19]. When disinhibited by developmental cues, CED-4 activates the *C. elegans* executioner caspase CED-3 [20], [21]. In the engulfing cell, several partially redundant pathways govern the membrane and cytoskeletal rearrangements required for phagocytosis of the dying cell (Figure S1) [22]–[25].

*C. elegans* was instrumental in illuminating the core features of programmed cell death and clearance because it is highly amenable to genetic and experimental manipulation. Recently, we implicated the human disease gene progranulin in the regulation of programmed cell death using a *C. elegans* mutant [26]. The regulation and function of progranulin are particularly interesting because of its links to disease. Progranulin haploinsufficiency causes the human neurodegenerative disease frontotemporal lobar degeneration while homozygous null carriers develop neuronal ceroid lipofuscinosis [27]–[29]. Allelic variations in the gene have also been linked to Alzheimer Disease, Parkinson Disease and amyotrophic lateral sclerosis (ALS) [30]–[34], and altered progranulin levels have been implicated in autoimmune disease [35], [36], cancer [37]–[42] and ischemic injury [43], [44]. Thus, precise regulation of progranulin levels is important for maintaining health and homeostasis.

Previously, we showed that progranulin normally functions to regulate the rate of apoptotic cell engulfment during the process of programmed cell death [26]. In *pgrn-1(-)* mutants, apoptotic cells are cleared approximately twice as fast as normal. We also showed that macrophages from progranulin null-mutant mice are able to engulf apoptotic cells more rapidly than are wild-type macrophages. Thus, progranulin, like *mtm-1*, *abl-1* and *srgp-1*, is a negative regulator of programmed cell death clearance [25], [26], [45]–[47] (Figure S1).

Given the close relationship between environmental stress and age-related disease, we asked whether *pgrn-1(-)* mutants exhibited an altered response to cellular stressors. We found that they did. However, unexpectedly, they demonstrated *increased* stress resistance. Even more surprisingly, we found that the same was true of mutations that perturb cell death in other ways, suggesting that a stress response pathway is activated when any part of the programmed cell death pathway does not proceed normally. Our findings reveal an unexpected link between mechanisms that control life-or-death decisions at the level of the individual cell and at the level of the entire animal.

## Results

### Loss of *pgrn-1* Confers Stress Resistance

**Figure 1:**
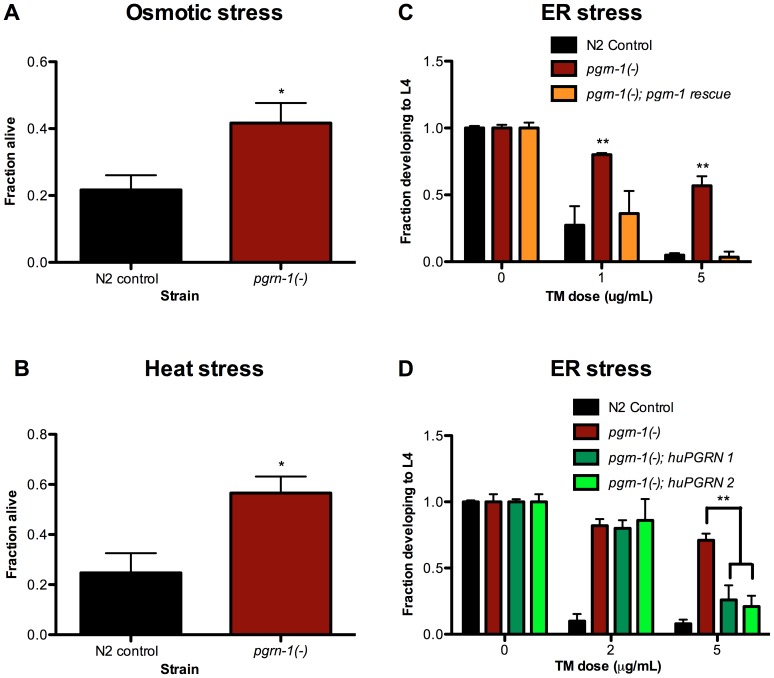
*pgrn-1(-)* mutants are resistant to osmotic, heat and ER stress. N2 control and *pgrn-1(tm985)* animals were subjected to various stressors and then scored for survival or ability to develop from egg to L4 stage. (A) Day 1 adult animals were treated with 600 mM NaCl for 24 hours and scored for survival (Student's t test). (B) Day 1 adult animals were incubated at 35°C for 8 hours and scored for survival (Student's t test). (C) Embryos from WT, *pgrn-1(-)* or *pgrn-1(-)* expressing a *C. elegans* progranulin rescue construct were treated with 0, 1 or 5 µg/mL tunicamycin (TM) for 3 days to induce ER stress and scored for ability to develop to the L4 stage (Two-way ANOVA with Bonferroni post-tests). (D) Embryos from WT, *pgrn-1(-)* or *pgrn-1(-)* expressing a human progranulin rescue construct (two independently integrated and outcrossed strains) were treated with TM as in (C) (Two-way ANOVA with Bonferroni post-tests). Results shown are representative of at least 2 experiments. Error bars represent standard deviation. Statistical comparisons here are to N2 control. n.s. not significant, *p<0.05, **p<0.01, ***p<0.001. For additional statistical data, please see Table S1A–D.

We tested the resistance of progranulin mutants to several environmental stressors. We found that compared to wild-type controls, *pgrn-1(tm985)* mutants were resistant to osmotic, heat and endoplasmic reticulum (ER) stress (the latter as measured by resistance to tunicamycin, an inhibitor of N-linked glycosylation) (Figure 1A–C, Tables S1A–C). In contrast, *pgrn-1* mutants had normal responses to oxidative stress (paraquat), genotoxic stress (UV light) and pathogen exposure (*P. aeruginosa* and *S. enterica*; Figure S2A–C and data not shown). Reintroducing either the *C. elegans* or human progranulin gene into *pgrn-1(-)* mutants rescued or partially rescued the mutant stress resistance phenotypes (Figure 1C–D, Tables S1C–D). The partial rescue by human progranulin only at higher doses of tunicamycin could be due to species differences or differences in binding affinities to the progranulin receptor.

What do heat, osmotic stress and tunicamycin have in common? One possibility is that they all beget unfolded proteins and induce the ER unfolded protein response. To address this idea, we tested the ability of each stressor to increase expression of *hsp-4*. HSP-4 is the nematode ortholog of mammalian *grp78/*BiP/HSP70, and is upregulated by heat and ER stress [48], [49]. We confirmed that heat stress and tunicamycin increased *P_hsp-4_::gfp* reporter levels (Figure S3A–B), and found that paraquat, UV irradiation and exposure to *P. aeruginosa* did not (Figure S4A–C). However, under our conditions, osmotic stress did not increase *P_hsp-4_::gfp* levels (Figure S3C). Thus, induction of the ER stress-resistance marker *P_hsp-4_::gfp* is not a feature that unifies heat, tunicamycin and osmotic stress.

### Apoptosis-Defective Mutants Are Stress Resistant

**Figure 2:**
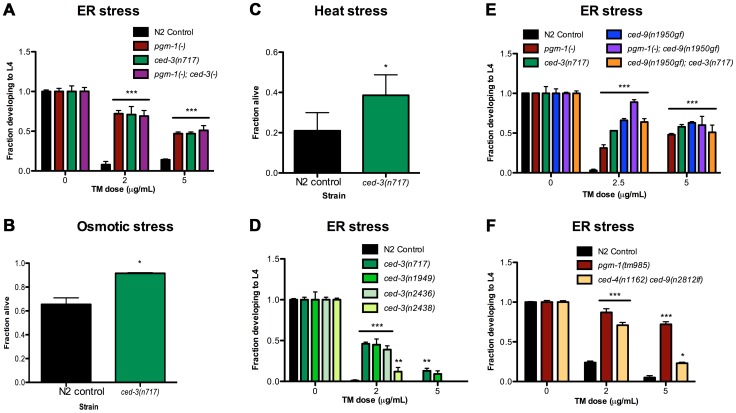
Mutations in programmed cell death genes *ced-3* and *ced-9* confer stress resistance. (A) *ced-3(n717)* animals were subjected to ER stress with indicated doses of tunicamycin (TM) and then scored for the ability to develop from egg to L4 stage. (B–C) Day 1 adult *ced-3(n717)* animals were exposed to 600 mM NaCl for 24 hours or thermal stress at 35°C for 8 hours and scored for survival. (D) Strong (*n717*), moderate (*n1949, n2436*) or weak (*n2436*) alleles of *ced-3* were tested for response to ER stress. (E) A *ced-9(n1950)* gain-of-function allele was tested for ER stress resistance in wild-type, *pgrn-1(-)* or *ced-3(-)* backgrounds. *(F) ced-4(n1162)* and *ced-9(n2812)* loss-of-function mutants were treated with TM and scored for the ability to develop from egg to L4 stage. Results shown are representative of at least 2 experiments except for (C), which is an average of 3 experiments. Error bars represent standard deviation. Statistical comparisons are to N2 control (Student's t test or ANOVA with Bonferroni post-tests). n.s. not significant, *p<0.05, **p<0.01 ***p<0.001. For additional statistical data, see Table S2A–D.

Because loss of function of *pgrn-1*, a regulator of programmed cell death clearance, caused stress resistance, we asked whether other mutations affecting programmed cell death would also affect the stress response of the whole animal. In contrast to progranulin mutants, loss-of-function mutations in the gene encoding the executioner caspase *ced-3* prevent apoptosis [15]. In *ced-3* loss-of-function mutants, cells that normally die during development instead persist. Surprisingly, we found that *ced-3(n717)* mutant animals also exhibited increased resistance to ER stress (Figure 2A and Tables S2A). Moreover, *pgrn-1(-); ced-3(n717)* double mutants were no more stress-resistant than were either of the single mutants (Figure 2A and Table S2A), suggesting that *pgrn-1* and *ced-3* mutations may activate the same stress response pathway. *ced-3(n717)* mutants could also exhibit osmotic and heat stress resistance, albeit not as consistently as ER stress resistance (Figure 2B–C and Table S2B). Like *pgrn-1* mutants, *ced-3(n717)* mutants did not exhibit resistance to paraquat or UV light (Figure S5).

Many alleles of *ced-3* have been isolated, and they form an allelic series based on their ability to inhibit programmed cell death [50]. We found that *ced-3* alleles exhibited graded levels of ER stress resistance that were correlated with their ability to block programmed cell death. The strong *ced-3* allele *n717* was more resistant to ER stress than were two intermediate strength (*n1949* and *n2436*) alleles, and these, in turn, were more resistant than the weak (*n2438*) allele (Figure 2D and Table S2A). These results are consistent with a model in which the stress resistance conferred by *ced-3* mutations is mechanistically related to the apoptotic killing conferred by *ced-3*.

In *C. elegans*, *ced-9* and *ced-4* regulate the ability of *ced-3* to activate programmed cell death [17]. The Bcl-2-like protein CED-9 inhibits CED-4/Apaf1 activity, blocking cell death; whereas activated CED-4 cleaves CED-3 and activates its caspase function, leading to cell death [18], [51]. Therefore, *ced-9 gain-of-function* (*gf*) and *ced-4 loss-of-function (lf* or *-)* mutations are similar to *ced-3(-)* mutations in the sense that they impair programmed cell death, whereas *ced-9(lf)* mutations cause excessive cell death (and animal lethality) due to uncontrolled activity of CED-4 and CED-3. We tested *ced-9(n1950gf)* and *ced-4(n1162lf)* single mutants, as well as a *ced-4(n1162lf) ced-9(n2812lf)* double mutant, for their responses to ER stress. We found that *ced-9(gf)* mutants were resistant to ER stress (Figure 2E and Table S2C). Further, the stress resistance conferred by *ced-9(gf)* mutations was not additive with that conferred by either *pgrn-1(-)* or *ced-3(-)* mutations, once again suggesting that these genes affect the same stress-response pathway (Figure 2E, Table S2C). We also found that *ced-4(lf)* single mutants were resistant to ER stress at low doses of tunicamycin in one of two experiments (Table S2D), and that these mutants did not require intact *ced-9* for this stress resistance (Figure 2F, Table S2D). These findings suggest that *ced-4* (and likely *ced-3*) may be genetically downstream of *ced-9* in the stress-resistance pathway, as it is in the cell death pathway. However, since *ced-4(lf)* animals displayed an incomplete degree of stress resistance compared to *ced-3(lf)* and *ced-9(gf)* mutants, it remains possible that *ced-4* is dispensable or redundant in this stress response pathway.

### Inhibition of Apoptotic Cell Engulfment Also Confers Stress Resistance

**Figure 3:**
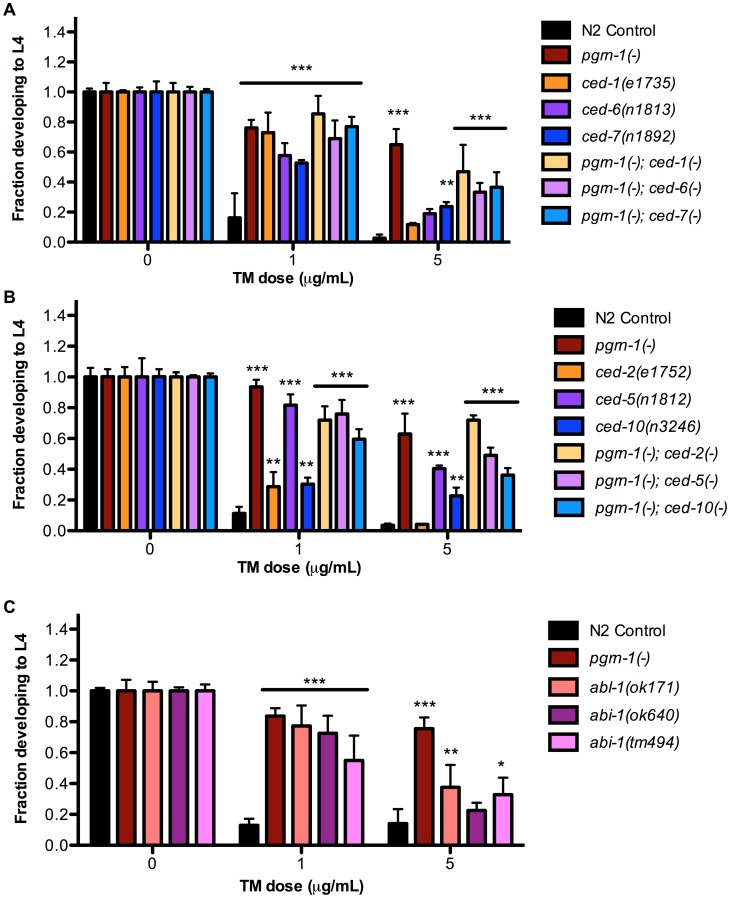
Engulfment mutations enhance ER stress resistance. (A) Mutations in *ced-1(e1735), ced-6(n1813)* and *ced-7(n1892)* with or without *pgrn-1(tm985)* in the background were tested for ER stress resistance by tunicamycin (TM) treatment. (B) Mutations in *ced-2(e1752), ced-5(n1812)* and *ced-10(n3246)* with or without *pgrn-1(tm985)* in the background were tested for ER stress resistance by tunicamycin treatment. (C) Mutations in *abl-1(ok171), abi-1(ok640)* and *abi-1(tm494)* were tested for ER stress resistance by tunicamycin treatment. Results shown are representative of at least 2 experiments except in the case of (C) which was performed once. Error bars represent standard deviation. Statistical comparisons are to N2 control (ANOVA with Bonferroni post-tests). n.s. not significant, *p<0.05, **p<0.01, ***p<0.001. For additional statistical data, see Table S3A, B, and D.

In addition to mutations that accelerate the clearance of apoptotic corpses, or prevent apoptosis altogether, we also asked whether mutations in apoptotic cell engulfment pathways affected stress resistance. We found that certain engulfment mutant alleles increased ER stress resistance, although the degree of resistance seen in engulfment mutants was generally less than that seen in animals carrying *pgrn-1(-)* or strong *ced-3(-)* mutant alleles. The engulfment mutants *ced-1(e1735), ced-6(n1813)*, *ced*-*7(n1892)* and *ced-2(e1752), ced-5(n1812), ced-10(n3246*) exhibited resistance to ER stress at low doses of tunicamycin (1 µg/mL). However they exhibited variable responses to tunicamycin at higher doses (5 µg/mL), with *ced-5*, *7* and *10* mutants demonstrating ER stress resistance but not *ced-1*, *2* or *6* mutants (Figure 3A–B and Table S3A–B). Once again, double mutants containing *pgrn-1* and engulfment mutations were not more resistant than *pgrn-1(-)* alone, suggesting that these mutations could potentially induce a common ER stress-resistance pathway (Figure 3A–B and Table S3A–B).

We also tested the response of engulfment mutants to other stressors. We found that *ced-1(e1735)* and *ced-2(e1752)* mutants were resistant to osmotic stress (Figure S6A and Table S3C). The *ced-2* mutant was also resistant to thermal stress in 1 of 2 trials (Figure S6 and Table S3C). As a group, the engulfment mutants were not as robustly resistant to environmental stressors as *pgrn-1* and *ced-3* mutants, which may be due to the partial functional redundancy of engulfment pathway genes (See Figure S1).

Like *pgrn-1*, the tyrosine kinase ABL-1 is a negative regulator of apoptotic corpse engulfment [45]. However, unlike *pgrn-1*, *abl-1* does not act through the canonical engulfment pathways. Instead, *abl-1* negatively regulates the engulfment gene *abi-1* to inhibit cell death clearance (See Figure S1). We asked whether these two genes might influence ER stress resistance, and found that both *abl-1* and *abi-1* mutants exhibited resistance to low doses of tunicamycin (Figure 3C, Table S3D). At higher doses of TM, *abl-1(-)* and one mutant allele of *abi-1, ok171*, were resistant to tunicamycin stress compared to wild type. Curiously, in some situations, *abl-1* mutations actually reduced ER stress resistance. For example, *abl-1(n1963)* mutations alone have no visible effect on engulfment of apoptotic corpses; however, *abl-1* mutations reduce the severity of the engulfment phenotype of *ced-1(n2091)* and *ced-6(n2095)* mutants [45]. Likewise, we found that *abl-1(n1963)* mutations reduced the level of ER stress resistance conferred by *ced-1(n2091)* and *ced-6(n2095)* mutations (Figure S7A and Table S3E). We do not have a simple unifying explanation for these findings at this time, but they indicate that *pgrn-1* is not the only negative regulator of cell engulfment that can affect ER stress resistance.

We also tested two additional genes that may modulate but are not directly involved in programmed cell death for stress response phenotypes. A mutation in *unc-73* enhances the effect of other engulfment mutants but alone has no engulfment defect [52]. A mutation in *unc-53* results in defective migration of cells and neuronal processes and the UNC-53 protein interacts with ABI-1 [53]. However, neither *unc-73(e936)* nor *unc-53(e404)* mutants exhibited ER stress resistance phenotypes (Figure S7B and Table S3F). Thus, not all genes involved in apoptotic cell engulfment are utilized for stress response.

### Stress Resistance in a Non-Apoptotic Programmed Cell Death Pathway

Recently, *pqn-41* was identified as a mediator of a type of non-apoptotic cell death [54]. This type of cell death, characterized by crenellation of the nuclear envelope and organelle swelling, occurs independently of the *ced-3* caspase and engulfment genes [55], [56]. To determine if *pqn-41* affects ER stress resistance, we tested a deletion mutant, *ns924*. We found that *pqn-41(ns924)* mutants were resistant to ER stress at a low dose of tunicamycin but not at a high dose, similar to some of the engulfment mutants we tested such as *ced-1*, *ced-2* and *ced-6* (Figure S7C and Table S3G). These data suggest that organismal stress resistance may be linked to both apoptotic and non-apoptotic programmed cell death.

Together, these findings indicate that perturbing *C. elegans* programmed cell death in a variety of ways, either by affecting the initiation of cell death or the engulfment of the dying cell, can confer whole-animal resistance to environmental stress.

### The Unfolded Protein Response (UPR) Gene *ire-1* Mediates ER Stress Resistance of *pgrn-1* Mutants

**Figure 4:**
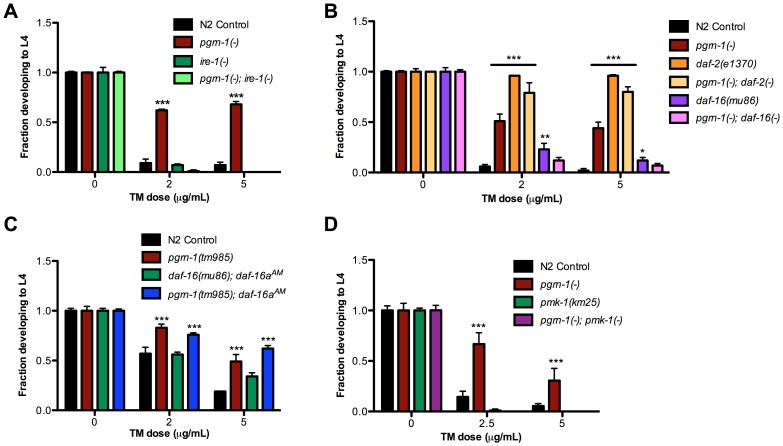
*pgrn-1(-)* resistance to ER stress may be partially dependent on the UPR pathway, *daf-16* and *pmk-1*. (A) Embryos from the indicated strains were grown on plates with tunicamycin (TM) and assessed for their ability to develop to the L4 stage. (B) *daf-2(e1370)* and *daf-16(mu86)* were tested for ER stress resistance with and without *pgrn-1(-)* in the background. (C) *daf-16(mu86); muIs113* and *pgrn-1(tm985); muIs113* animals were tested for ER stress resistance. (D) *pmk-1(km25)* and *pgrn-1(-); pmk-1(-)* mutants were tested for ER stress resistance. Error bars represent standard deviation. Statistical comparisons are to N2 control (Two-way ANOVA with Bonferroni post-tests). *p<0.05, **p<0.01, ***p<0.001. For additional statistical data, see Table S4A–D.

Because of recent findings connecting the unfolded protein response with neurodegenerative diseases [57], we decided to investigate the mechanism by which a *pgrn-1* mutation affected ER stress resistance. The UPR is the cellular program that responds to ER stress. The UPR is mediated by three ER resident proteins encoded by *ire-1*, *pek-1* (mammalian Perk) and *atf-6* [58]. Mutations in these genes impair the response to ER stress in *C. elegans* [59], [60]. Part of this stress response includes alternative splicing of *xbp-1* mRNA by activated IRE-1 and the consequent upregulation of XBP-1 target genes, such as *hsp-4*, the nematode ortholog of mammalian *grp78/*BiP/HSP70 [48], [49]. To investigate the role of the UPR in the stress resistance of apoptosis mutants, we first tested whether *pgrn-1* mutants required *ire-1* for ER stress resistance. We tested a *pgrn-1; ire-1* double mutant and found that resistance in *pgrn-1* mutants was dependent on *ire-1* (Figure 4A and Table S4A) suggesting that the mechanism of stress resistance of cell death mutants requires this branch of the UPR pathway. One possibility was that the IRE-1 pathway is constitutively activated in *pgrn-1* mutants. To test this, we measured the levels of spliced *xbp-1* mRNA. Interestingly, we found no changes in the levels of spliced *xbp-1* mRNA in *pgrn-1* mutants compared to wild type (Figure S8). We also investigated whether *ced-3* or *ced-1* mutants exhibited increased levels of spliced *xbp-1* mRNA. Similar to *pgrn-1* mutants, they did not (Figure S8).

Since the *hsp-4* gene is a target of active XBP-1, we measured whether *pgrn-1* mutants displayed increased *P_hsp-4_::gfp* reporter levels. We found that except for one time point at the L4 stage of larval development, *P_hsp-4_::gfp* levels in *pgrn-1* mutants were largely unchanged compared to controls. Correspondingly, levels of *P_hsp-4_::gfp* in *ced-3(n717)* mutants were also generally unchanged compared to controls (Figure S9). These data suggest that although *pgrn-1* mutations affect the ER stress response through the *ire-1* gene, the downstream splicing of *xbp-1* mRNA and expression of *P_hsp-4_::gfp* is not affected. *pgrn-1* mutations may somehow make the IRE-1 branch of the UPR more effective without dramatically changing its activity.

### The FOXO Transcription Factor DAF-16 and the MAPK PMK-1 Are Required for ER Stress Resistance of *pgrn-1* Mutants

Mammalian progranulin has been demonstrated to activate the insulin/IGF-1 pathway and downstream MAP kinases [37], [61], [62]. Animals carrying mutations in the *C. elegans* insulin/IGF-1 receptor, *daf-2*, are long-lived [63], [64] and resistant to many stressors, including heat, osmotic stress and ER stress [63], [64]. The longevity and stress resistance of *daf-2* mutants require the FOXO transcription factor *daf-16* [60]. Upon inactivation of *daf-2*, DAF-16 accumulates in the nucleus [65] where it regulates transcription of stress response genes. We found that the degree of ER stress resistance of *pgrn-1(tm985); daf-2(e1370)* double mutants was similar to that of *daf-2(-)* single mutants (Figure 4B and Table S4B). *pgrn-1* mutants also required intact *daf-16* for stress resistance, as *daf-16(mu86) pgrn-1(tm985)* double mutants were no more stress resistant than were single *daf-16(mu86)* mutants (Figure 4B and Table S4B). These findings suggest that *pgrn-1* may be part of the *daf-2* pathway or act with *daf-2* to confer stress resistance. However, unlike mutations in *daf-2, pgrn-1(-)* does not affect nuclear localization of DAF-16::GFP protein (Figure S10). DAF-16::GFP localization is also unaffected in *ced-1* and *ced-3* mutant animals (Figure S10). To determine if nuclear localization of DAF-16 would further increase stress resistance in *pgrn-1* mutants, we crossed the *daf-16a^AM^* transgene (which causes DAF-16 nuclear accumulation due to mutation of its AKT-phosphorylation sites) into a *pgrn-1(-)* background [65]. We found that a *pgrn-1(-); daf-16a^AM^* strain was no more stress resistant than was the *pgrn-1* mutant alone (Figure 4C and Table S4C).

Others have shown that adult-only *ced-3* RNAi extends lifespan without altering DAF-16::GFP localization [66]. Given the correlation between lifespan extension and some forms of stress resistance [67], we tested the lifespan of *ced-3*, *ced-1* and *ced-2* mutants. In earlier work, we showed that *pgrn-1(-)* mutant lifespan is no different than wild type [26]. Whereas a *ced-3(-)* mutation significantly extended lifespan compared to wild type, *ced-1* and *ced-2* mutations did not (Figure S11), indicating that longevity and this type of organismal stress resistance can be dissociated.

Several MAP kinases are required for responses to cellular stressors in *C. elegans*. The PMK-1/p38 MAP kinase encoded by *pmk-1* is required for resistance to oxidative stressors [68], pathogenic bacteria [69] and exogenously induced ER stress [70]. We confirmed that *pmk-1* mutations increased sensitivity to ER stress and found that *pgrn-1(-); pmk-1(km25)* double mutants were no more resistant to ER stress than were *pmk-1* single mutants. Thus, *pmk-1* is required for the ER stress resistance induced by *pgrn-1* mutations (Figure 4D and Table S4D).

Progranulin is a secreted protein. In mammals, two progranulin receptors have been identified, the tumor necrosis factor receptor (TNFR) and sortilin [71]. Thus, we tested a downstream TNF receptor associated factor (TRAF) mutant, *trf-1(nr2014)*, for stress resistance and epistasis with *pgrn-1(-)*. *trf-1* mutants were not stress resistant compared to wild type and *pgrn-1(-)* did not require *trf-1* for its stress resistance (Figure S12A). We also tested two mutant alleles of *trk-1*, a *C. elegans* neurotrophin receptor similar to a co-receptor for sortilin, the other mammalian progranulin receptor. Again, *pgrn-1* mutants did not require *trk-1* for stress resistance (Figure S12B). Thus, an as yet unidentified receptor(s) appears to be required for progranulin to influence ER stress resistance in *C. elegans*.

### Apoptotic Cell Death Mutants Share Co-regulated Genes

**Figure 5:**
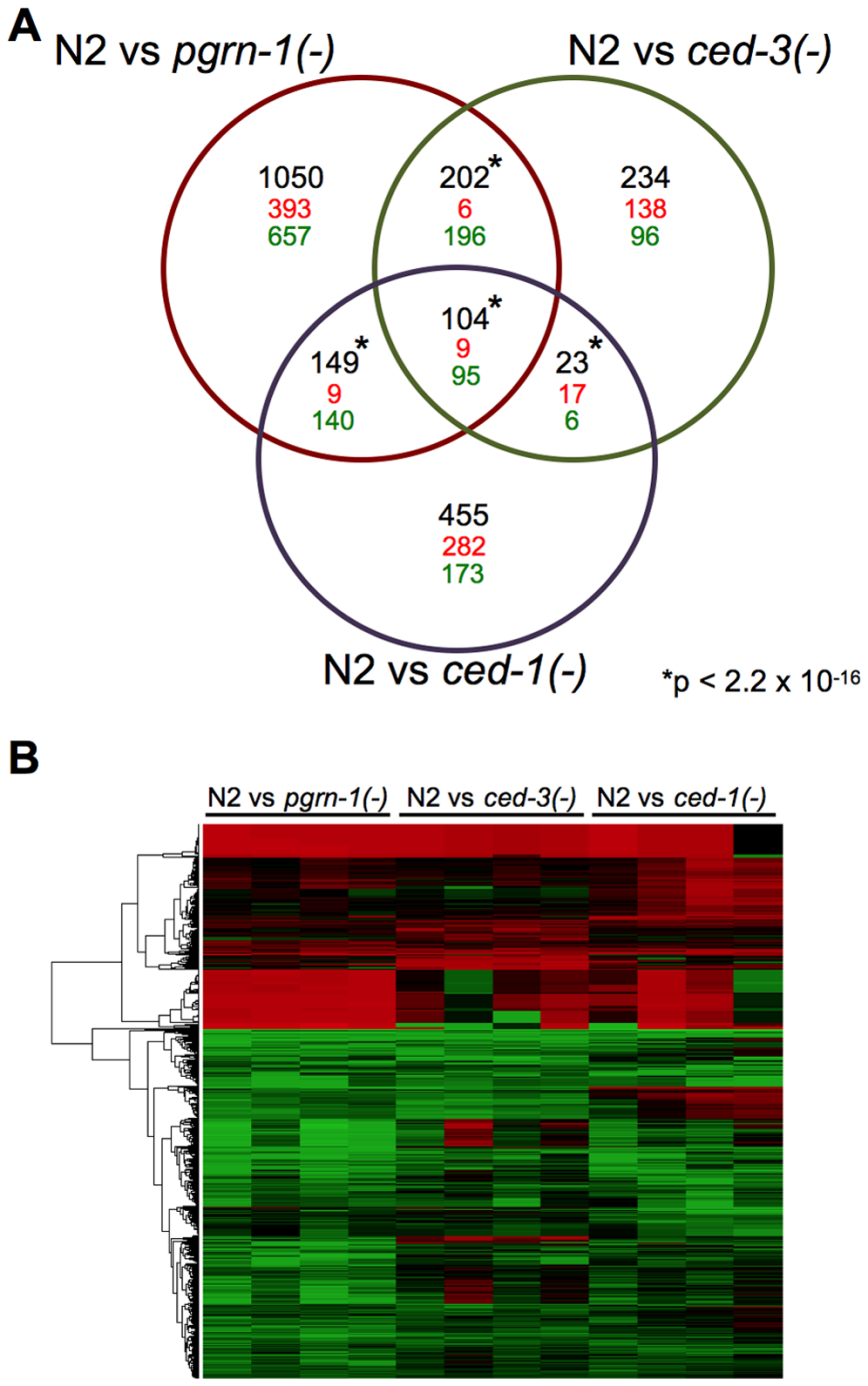
Programmed cell death mutants share differentially regulated genes. (A) Venn diagram of differentially regulated genes in day 1 adult wild-type (N2) animals versus *pgrn-1(tm985)*, *ced-3(n717)* or *ced-1(e1735)* mutants. Significance cut-off was an FDR of <0.05. The numbers in black represent the total number of overlapping genes, with direction of change (up-regulated = red, down-regulated = green) indicated below. The significance of the overlaps between the mutant strains was calculated using Fisher's exact test. See the Excel file (Table S5) for gene list. *p value<2.2×10^−16^. (B) Heat map depicting the fold changes of gene expression in *pgrn-1(tm985), ced-3(n717)* and *ced-1(e1735)* mutants compared to N2 wild-type animals for each of four biological replicates.

If mutations that perturb cell death in different ways act in the same stress-resistance pathway, then they might share gene expression patterns that differ from wild type. To test this, we performed gene expression profiling by RNA sequencing (RNA-seq), comparing day 1 adult *pgrn-1(tm985)*, *ced-3(n717)* and *ced-1(e1735)* mutants to wild-type animals. This allowed us to assess 1) whether these strains have altered gene expression, and 2) whether their differentially expressed genes are shared, suggesting the involvement of a common pathway. In spite of different cell death phenotypes of these mutants, RNA-seq revealed that all three mutants down-regulated the same 95 genes and up-regulated the same 9 genes, a highly significant portion of the total transcriptome (p<10^−16^) (Figure 5 and Table S5). Of the genes that share differential regulation in our mutants, a significant number are regulated by DAF-16 (Table S5). These findings are consistent with the possibility that the stress resistance phenotypes of these three mutants may be due to the involvement of shared pathways.

## Discussion

We have shown that mutations that impair *C. elegans* programmed cell death in any of three ways—by inhibiting apoptosis, by impairing corpse clearance or by accelerating corpse clearance—all enhance resistance to certain environmental stressors. These mutations do not confer resistance to all cellular stressors, as *pgrn-1* and *ced-3* mutants exhibit normal sensitivity to UV light and oxidative stress.

Several questions naturally follow from these findings. First, why are these mutants resistant to specific stressors? Tunicamycin is an N-linked glycosylation inhibitor that causes retention of translated proteins in the ER and induces the unfolded protein response. Similarly, heat and osmotic stress cause ER and/or cytosolic proteins to unfold and activate signaling programs that induce expression of heat shock proteins and other chaperones. Thus, it seems possible that cell death mutations all trigger a response specific for unfolded proteins, such as the ER unfolded protein response. Consistent with this, we found that the cell-death-related stressors tunicamycin and heat both activated the ER stress-response gene *hsp-4/*BIP, whereas the cell-death-unrelated stressors *Pseudomonas*, UV and paraquat did not. However, *P_hsp-4_::gfp* was not induced by osmotic stress and *pgrn-1(-)* mutation did not increase basal *P_hsp-4_::gfp* expression levels. Thus, defects in cell death can do more to protect the animal than simply to induce the canonical UPR. They must generate a more multifaceted response that can maintain proteostasis.

The mechanism by which cell death mutations induce animal-wide stress resistance is not known. If different cell-death mutations affected different pathways, or the same pathway to different extents, then one would expect double mutants to be even more stress resistant than the individual single mutants. As this was not the case, it is possible that all of these mutations trigger the same stress response, though other interpretations remain possible. We identified some of the genes required for *pgrn-1* mutants to resist the ER stressor tunicamycin. We found that this stress resistance requires an intact *ire-1* gene, the MAP kinase PMK-1 and the transcription factor DAF-16/FOXO. We also identified a number of genes that are differentially regulated by all three of our mutants (compared to wild type), suggesting that they may achieve stress resistance by recruiting shared genes and/or pathways. It will be very interesting to explore these shared genes in future studies.

In contrast to the selective stress resistance of *pgrn-1* mutants, *daf-2* mutants are resistant to most environmental stressors. Thus, decreased DAF-2/insulin/IGF-1 signaling activates a genetic program that more generally elevates organismal resilience. *daf-2* mutants appear to increase their resistance to ER stress by making the *ire-1/xbp-1* pathway more efficient, possibly by activating stress-response transcription factors like DAF-16 that collaborate with *ire-1/xbp-1* to induce new protective genes [60]. In concordance with this, *daf-2* mutants actually have reduced levels of expression of *ire-1/xbp*-1-regulated genes such as *hsp-4*/BIP. While this was not the case for *pgrn-1* or *ced-3* mutants, whose *P_hsp-4_::gfp* levels were not decreased, there are some unexpected similarities between the ER stress-resistance phenotypes of cell-death mutants and *daf-2* mutants. First, the ER stress resistance phenotypes of *pgrn-1* and *daf-2* mutants require *daf-16*, either fully (*pgrn-1* mutant) or partially (*daf-2* mutant). Second, both ER stress responses are completely dependent on *ire-1*, yet in neither case is *xbp-1* splicing increased. Additionally, the cell death mutants exhibited either no increase or only slight increases in *P_hsp-4_::gfp* expression, rather than the substantial increase one might expect if this branch of the ER pathway were constitutively active. Finally, the *daf-2* and *pgrn-1* mutant ER stress-resistance phenotypes are not additive. Thus the ER-stress resistance pathways activated by these mutations likely share at least some mechanistic features.

It was striking that mutations that perturb programmed cell death in such different ways all had similar effects on environmental stress resistance. Why should mutations that lead to undead cells (apoptosis mutations), lingering corpses (engulfment mutations), and prematurely-engulfed corpses (progranulin mutations) all activate what appears to be (from genetic tests) the same stress resistance pathway? From an evolutionary perspective, this linkage may make sense. Presumably apoptosis evolved not only to sculpt tissues during development, but also to remove cells that are damaged and unable to perform their normal functions, or perhaps that are overtly harmful to the animal. Viewed in this way, impairments in programmed cell death could be interpreted by the organism as an inability to respond normally to stress. Perhaps, under these conditions, the animal uses an alternative, back-up system to survive; namely, the system we have described in this study. Specifically, animals could have developed a sensitive surveillance system that can detect abnormalities in cell death, and respond to them by activating another pathway that enhances their overall level of stress resistance. The existence of this type of alternative system could have increased animal fitness during evolution in turbulent or adverse environments.

While this model makes sense from an evolutionary perspective, other models are possible as well. For example, perhaps cell-death proteins, which act in a multi-step pathway to remove unwanted cells, also act together in a different pathway that has the effect of sensitizing the animal to various forms of stress. Non-cell death functions have, in fact, been described for cell-death effectors. In *C. elegans*, *ced-10(-)* mutations impair not only cell engulfment but also cell migration [72]. Another engulfment gene, *ced-1*, has also been implicated in neuronal regulation of innate immunity [73], [74]. In mammals, a defect in the BCL2-family protein BID impairs cytokine production in response to immune activation independently of its cell death signaling function [75]. Further, certain mammalian executioner caspases can activate microglia in response to inflammogens without causing microglial death [76]. Finally, in a mouse model of Alzheimer Disease, caspase activation may be responsible for tau cleavage and aggregate formation, thereby serving a protective function [77]. Thus, programmed cell death effectors could hypothetically act together to sensitize animals to certain stressors or, alternatively, to inhibit a stress-response pathway. In either case, perturbing programmed cell death would increase organismal stress resistance.

A protein in the flowering plant *Arabidopsis* may support the model that programmed cell death effectors can sensitize an animal to environmental stress. *Arabidopsis* can express a protein called RD21 that, like CED-3, is a cysteine protease, and, like progranulin, contains a granulin domain. Osmotic stress induces RD21 and, possibly as a consequence, leaf senescence. Interestingly, in response to stress RD21 undergoes a process of maturation in which its caspase domain cleaves and releases the granulin domain [78]–[80]. In RD21, the caspase and granulin domains are contained within the same molecule. However, perhaps in *C. elegans*, the two domains reside in different proteins but nevertheless act together to influence organismal stress resistance.

In summary, our findings indicate that programmed cell death effectors not only kill and remove individual cells, but also influence environmental stress resistance at the level of the whole animal. To our knowledge this is the first time that cell-death effectors like *ced-3, pgrn-1* and *ced-1* have been implicated in organismal stress resistance, and these findings raise many interesting new questions about both mechanism and evolution.

## Materials and Methods

### Strains

Unless otherwise indicated, *C. elegans* were cultured at 20°C using standard procedures [81]. Strains were kindly provided by the Mitani Laboratory (National Bioresource Project) at the Tokyo Women's Medical University and the Caenorhabditis Genetics Center (CGC) at the University of Minnesota. Strains were outcrossed four times to the laboratory N2 control strain (N2 Bristol). Descriptions of strains can be found at www.wormbase.org. The following strains were used:

AWK2 *ced-9(n1950gf) III; ced-3(n717) IV*

AWK74 *daf-16(mu86) I; ced-3(n717) IV*

AWK76 *daf-16(mu86) pgrn-1(tm985) I; muIs109[P_daf-16_::daf-16::gfp+P_odr-1_::RFP]*

AWK77 *daf-16(mu86) I; ced-3(n717) IV; muIs109[P_daf-16_::daf-16::gfp+P_odr-1_::RFP]*

AWK78 *ced-1(e1735) daf-16(mu86) I*

AWK80 *ced-1(e1735) daf-16(mu86) I; muIs109[P_daf-16_::daf-16::gfp+P_odr-1_::RFP]*

AWK109 *pgrn-1(tm985) I; pqn-41(ns294) III*

AWK111 *pgrn-1(tm985) I; muIs113[P_daf-16_::daf-16AM::gfp+rol-6]*

CB404 *unc-53(e404) II*

CB936 *unc-73(e936) I*

CF1037 *daf-16(mu86) I*

CF1041 *daf-2(e1370) III*

CF1934 *daf-16(mu86) I; muIs109[P_daf-16_::daf-16::gfp+P_odr-1_::RFP]*

CF2260 *N2; zcIs4[P_hsp-4_::gfp] V*

CF2473 *ire-1(ok799) II*

CF3050 *pgrn-1(tm985) I*

CF3165 *pgrn-1(tm985) I; zcIs4[P_hsp-4_-4::gfp] V*

CF3170 *pgrn-1*(tm985) *I*; *ire-1(ok799) II*

CF3196 *daf-16(mu86) pgrn-1(tm985) I*

CF3206 *pgrn-1(tm985) I*; *daf-2(e1370) III*

CF3324 *ced-3(n717) IV*

CF3419 *pgrn-1(tm985) I; ced-3(n717) IV*

CF3447 *pgrn-1(tm985) I; muIs189[P_pgrn-1_:: pgrn-1::polycistronic mCherry+P_odr-1_::CFP]*

CF3762 *ced-3(n2436) IV*

CF3656 *ced-2(e1752) IV*

CF3660 *ced-10(n3246) IV*

CF3662 *pgrn-1(tm985) I; ced-2(e1752) IV*

CF3667 *ced-1(e1735) I*

CF3672 *pgrn-1(tm985) ced-1(e1735) I*

CF3675 *pgrn-1(tm985) I; ced-10(n3246) IV*

CF3680 *ced-5(n1812) IV*

CF3683 *pgrn-1(tm985) I; ced-7(n1892) III*

CF3684 *pgrn-1(tm985) I; ced-5(n1812) IV*

CF3685 *pgrn-1(tm985) I; ced-6(n1813) III*

CF3687 *pgrn-1(tm986) I; muIs211[P_egl-3_::huPGRN::polycistronic mCherry+P_odr-1_::CFP] Line 1*

CF3688 *pgrn-1(tm986) I; muIs211[P_egl-3_::huPGRN::polycistronic mCherry+P_odr-1_::CFP] Line 2*

CF3762 *ced-3(n1949) IV*

CF3802 *ced-3(n717) IV; zcIs4[P_hsp-4_::gfp] V*

CF3808 *trk-1(tm3985) X*

CF3809 *trk-1(tm4054) X*

CF3817 *pgrn-1(tm985) I; trk-1(tm3985) X*

CF3818 *pgrn-1(tm985) I; trk-1(tm4054) X*

CF3821 *trf-1(nr2014) III*

CF3833 *pgrn-1(tm985) I; trf-1(nr2014) III*

CF3879 *pgrn-1(tm985) I; pmk-1(km25) IV*

CF3881 *pgrn-1(tm985) I; ced-9(n1950gf) III*

FX494 *abi-1(tm494) III*

KU25 *pmk-1(km25) IV*

MT2547 *ced-4(n1162) III*

MT4433 *ced-6(n1813) III*

MT4982 *ced-7(n1892) III*

MT4770 *ced-9(n1950gf) III*

MT7384 *ced-4(n1162) ced-9(n2812lf) III*

MT16077 *ced-1(n2091) I; abl-1(n1963) X*

MT19956 *ced-6(n2095) III; abl-1(ok171) X*

OS4023 *pqn-41(ns294) III*

RB829 *abi-1(ok640) III*

XR1 *abl-1(ok171) X*

### Generation of Transgenic *C. elegans* Strains

To generate a *C. elegans pgrn-1* rescue construct, full-length *pgrn-1a*+TAA stop codon and its endogenous 0.5 kB upstream promoter were cloned into a Gateway polycistronic mCherry vector (courtesy K. Ashrafi lab, UCSF). The resulting plasmid (*P_pgrn-1_::pgrn-1+TAA::polycistronic mCherry*) expresses both progranulin and mCherry and functions as a full length rescuing construct when expressed in the *pgrn-1* mutant. To generate a human progranulin rescue strain, the pan-neuronal *egl-3* promoter (courtesy of K. Ashrafi lab) and the human progranulin cDNA sequence were cloned into a Gateway polycistronic mCherry vector (*P_egl-1_::human PGRN::polycistronic mCherry*).

The constructs were microinjected separately into the gonads of day 1 adult *C. elegans*. Stable monogenic lines were isolated and analyzed using Leica fluorescent, Zeiss Axioplan 2 or Nikon Spectral Confocal microscopes. Extrachromosomal arrays were integrated by UV irradiation by the method of C. Frank *et al.* [82] and outcrossed at least 5 times to our lab's wild-type N2 control strain.

### Stress Assays

For thermal and osmotic stress assays on Day 1 worms, L4-stage animals were picked and grown at 20°C overnight. For thermal stress assays, worms were moved to a 35°C incubator for 12 hours and then scored for survival. Osmotic stress assays were performed by the method of Lamitina *et al.* [83] with the following modifications: worms were fed OP50 bacteria, worms were cultured at 20°C prior to the assay, and assays were performed on NG-based plates at 20°C with increasing amounts of NaCl added as indicated. For paraquat stress assays, individual animals were placed in 96-well plates with 100 µL of 250 µM methyl viologen (paraquat, Sigma-Aldrich) dissolved in M9 and scored for movement every 1 hour at 25°C. For genotoxic stress assays, day 1 adult animals were transferred to unseeded plates and treated with 1200 J/m^2^ UV light in a Stratalinker 1800 (Stratagene). Animals were then scored for survival every 24 hours. Pathogen stress was performed by transferring worms to plates seeded with *P. aeruginosa* or *S. enterica* starting at day 1 and scoring each subsequent day for survival. In all assays, animals that failed to move in response to a gentle touch with a metal pick were scored as dead.

For ER stress assays, synchronized eggs were transferred to plates containing 0, 1, 2 or 5 µg/mL of tunicamycin (EMD Chemicals). After 3 days, animals that developed to the L4 stage were quantified. Figures show fraction of animals that develop to L4 stage normalized to percent hatching on 0 µg/mL tunicamycin for each strain.

Statistical analyses were performed in GraphPad Prism statistical package with tests as indicated in figure legends.

### Lifespan Analysis

Wild-type, *ced-1(e1735)*, *ced-2(e1752)* and *ced-3(n717)* strains were grown at 20 degrees Celsius (C), then picked to fresh OP50 at the L4 stage and shifted to 25 degrees C. Subsequent lifespan analysis was done at this temperature. Animals were transferred every day to fresh plates until progeny production ceased. Animals that crawled off the plate, exploded, bagged, or became contaminated were censored. GraphPad Prism was used to calculate mean life spans and perform statistical analyses. *P* values were determined using log-rank (Mantel-Cox) statistics.

### *xbp-1* RT-PCR

*C. elegans* eggs were obtained by bleaching, then plated onto *E. coli* OP50 and allowed to develop at 20°C to day 1 of adulthood. At this point, positive controls were exposed to 5 mg/ml tunicamycin for 5 hours while all other worms were left untreated. After washing animals off plates, Trizol was added and samples were frozen in liquid nitrogen. Animals were lysed in a Mini-Beadbeater (Biospec products) for 10 minutes at the maximal setting. Total RNA was isolated using a phenol/chloroform extraction and DNA contamination was removed with DNA-free treatment (Ambion). cDNA was synthesized (iScript) using oligo (dT) primers and RT-PCR was performed using primers that amplify an ∼200 bp unspliced transcript and an ∼180 bp spliced transcript. Forward primer sequence: 5′ ctacgaagaagaagtcgtcgg 3′ and reverse primer sequence: 5′ ttcttgttgcgatccatgtg 3′. RT-PCR products were analyzed by running them out on a 3% agarose gel stained with ethidium bromide.

### Quantification of Fluorescence

Animals expressing the *P_hsp-4_::gfp* transgene were anaesthetized on agarose pads containing 2.5 mM levamisole. Whole worm images were taken using a Retiga EXi Fast1394 CCD digital camera (QImaging, Burnaby, BC, Canada) using the 5× objective on a Zeiss Axioplan 2 compound microscope (Zeiss Corporation, Germany). Each image was taken so that the intestine was in focus and exposure time was calibrated to minimize number of saturated pixels for the set of animals. Images within each experiment were acquired using identical settings and exposure times to allow direct comparisons. Fluorescence intensity was measured by outlining the entire worm. Openlab 4.0.2 software (Improvision, Coventry, UK) was then used to quantify total intensity of each pixel in the selected area. Measurements were obtained by subtracting the minimum intensity from the mean intensity and taking the average of these calculations for 8–10 animals per time-point.

### RNA-Seq Analysis

Total RNA was isolated from each of the strains *pgrn-1(tm985), ced-3(n717), ced-1(e1735)* and wild-type (N2E) using a phenol/chloroform extraction, and DNA contamination was removed with DNA-free treatment (Ambion). Samples were extracted in quadruplicates (four biological replicates for each strain), for a total of 16 samples. Total RNA was quantified using the RiboGreen assay and RNA quality was checked using an Agilent Bioanalyzer (Agilent). RNA Integrity Numbers (RINs) were >8 in all the samples. Libraries for RNA-seq were prepared using the Illumina TruSeq library preparation protocol (Illumina Inc), multiplexed into a single pool and sequenced using an Illumina HiSeq 2500 sequencer across 4 lanes of 2 Rapid Run SR 1×50 flow cells. After demultiplexing, we obtained between 13 and 32 million reads per sample, each one 50 bases long. Quality control was performed on base qualities and nucleotide composition of sequences. Alignment to the *C. elegans* genome (ce10) was performed using the STAR spliced read aligner (PMID 23104886) with default parameters. Additional QC was performed after the alignment to examine the following: level of mismatch rate, mapping rate to the whole genome, repeats, chromosomes, and key transcriptomic regions (exons, introns, UTRs, genes). Between 92 and 93% of the reads mapped uniquely to the worm genome. Total counts of read-fragments aligned to candidate gene regions within the *C. elegans* reference gene annotation were derived using HTS-seq program and used as a basis for the quantification of gene expression. Only uniquely mapped reads were used for subsequent analyses. Following alignment and read quantification, we performed quality control using a variety of indices, including sample clustering, consistency of replicates, and average gene coverage. Differential expression analysis was performed using the EdgeR Bioconductor package (19910308), and differentially expressed genes were selected based on False Discovery Rate (FDR Benjamini Hochberg adjusted p- values) estimated at ≤5%. Clustering and overlap analyses were performed using Bioconductor packages within the statistical environment R (www.r-project.org/). Gene Ontology annotation was performed using DAVID (david.abcc.ncifcrf.gov/). DAF-16 dependent genes were curated from published reports [84], [85] and Wormmart annotation (http://caprica.caltech.edu:9002/biomart/martview).

## Supporting Information

Figure S1 Genetic pathways that regulate programmed cell death in apoptotic and engulfing cells in *C. elegans*. Genes that normally promote cell death and engulfment are in green while those that normally inhibit them are shown in red. Genes tested in this study are in bold.
(TIFF)

Figure S2 Related to Figure 1. A mutation in *pgrn-1* does not confer oxidative, UV or pathogen stress resistance. Day 1 adult N2 or *pgrn-1(tm985)* animals were subjected to oxidative stress with 250 µM paraquat (A), genotoxic stress with 1200 J/m^2^ UV light (B) or pathogen stress by feeding *P. aeruginosa* (C). Worms were scored for survival at indicated times and analyses by log-rank Mantel-Cox test were performed. (A) p = 0.1, N>30 animals/strain. (B) p = 0.054, n>50 animals/strain. (C) p = 0.078, N>50 animals/strain. Results shown are representative of at least 2 experiments except in A, which was performed 3 times with p values of 0.0034, 0.01 and 0.125.
(TIFF)

Figure S3 Related to Figure 1. Heat and tunicamycin stress increase HSP-4::GFP levels while osmotic stress does not. (A) *P_hsp-4_::hsp-4::gfp* animals were incubated at 20°C (Control treated) or 35°C (Heat stress treated) for 4 hours then imaged on a Zeiss AxioImager. (B) *P_hsp-4_::hsp-4::gfp* animals were incubated at 20°C on agarose plates containing 0 µg/mL (Control treated) or 5 µg/mL (ER stress treated) tunicamycin. Twenty-four hours later, they were imaged on a Zeiss AxioImager. (C) *P_hsp-4_::hsp-4::gfp* animals were incubated at 20°C on agarose plates containing 51 mM NaCl (Control treated) or 400 mM NaCl (Osmotic stress treated). After 24 hours, they were imaged on a Zeiss AxioImager.
(TIFF)

Figure S4 Related to Figure 1. UV light, paraquat and *P. aeruginosa* do not increase HSP-4::GFP levels. (A) *P_hsp-4_::hsp-4::gfp* animals were mock-treated (Control treated) or exposed to 1200 J/m^2^ of ultraviolet light (UV stress treated). After 5 hours at 20°C, the animals were imaged on a Zeiss AxioImager. (B) *P_hsp-4_::hsp-4::gfp* animals were placed onto agarose plates with either OP50 bacteria (Control treated) or *P. aeruginosa* PA14 (Pathogen treated) bacteria. After incubation for 24 hours at 20°C, animals were imaged on a Zeiss AxioImager. (C) *P_hsp-4_::hsp-4::gfp* animals were placed in M9 solution containing either vehicle alone (Control treated) or 200 mM paraquat (Oxidative stress treated) for 3 hours. The animals that were still alive at that time were imaged on a Zeiss AxioImager.
(TIFF)

Figure S5 Related to Figure 2. A mutation in *ced-3* does not confer oxidative or UV stress resistance. Day 1 adult N2 or *ced-3(n717)* animals were subjected to oxidative stress with 250 µM paraquat (A) or genotoxic stress with 1200 J/m^2^ UV light (B). Worms were scored for survival at indicated times and analyses by log-rank Mantel-Cox test were performed. (A) p>0.05, N = 20 animals/strain. (B) p>0.05, N>35 animals/strain.
(TIFF)

Figure S6 Related to Figure 3. Mutations in engulfment genes confer resistance to osmotic and heat stresses. Day 1 adult *ced-1(e1735)* and *ced-2(e1752)* animals were exposed to 600 mM NaCl for 24 hours (A) or thermal stress at 35°C for 8 hours (B) and scored for survival. Error bars represent standard deviation. Statistical comparisons here are to N2 control (Student's t test or ANOVA with Bonferroni post-tests). *p<0.05, ***p<0.001. For additional statistical data, see Supplemental Table S3C.
(TIFF)

Figure S7 Related to Figure 3. ER stress resistance in related cell death mutants. A) Mutations in *ced-1(e2091)* and *ced-6(n2095)* with or without *abi-1(n2091* or *ok171*) in the background were tested for ER stress resistance by tunicamycin (TM) treatment. (B) Mutations in *unc-53(e404)* and *unc-73(e936)* were tested for ER stress resistance by tunicamycin treatment. (C) Mutations in *pqn-41(ns924)* with and without *pgrn-1(tm985)* in the background were tested for ER stress resistance by tunicamycin treatment. Results shown are representative of at least 2 experiments except in the case of (C) which was performed once. Error bars represent standard deviation. Statistical comparisons are to N2 control (ANOVA with Bonferroni post-tests). *p<0.05, **p<0.01, ***p<0.001. For additional statistical data, see Supplemental Tables S3E–G.
(TIFF)

Figure S8 Related to Figure 4. Levels of spliced *xbp-1* mRNA is unchanged in *pgrn-1(-)*, *ced-1(-)* or *ced-3(-)* mutants compared to wild type. Representative RT-PCR of *xbp-1* from RNA isolated from day 1 animals. Arrows point to the unspliced and spliced *xbp-1* bands and the loading control actin band. + TM refers to treatment with 5 mg/mL tunicamycin.
(TIFF)

Figure S9 Related to Figure 4. HSP-4 does not mediate stress resistance of *pgrn-1(-)* or *ced-3(-)* mutants. Baseline levels of HSP-4::GFP in wild-type, *pgrn-1(-)* and *ced-3(-)* animals. Animals expressing *P_hsp-4_::hsp-4::gfp* in an otherwise wild-type, *pgrn-1(tm985)* or *ced-3(n717)* background were synchronized by washing adults and larvae away from eggs. L1 animals were collected from hatched embryos 1 hour later while L2, L3 and L4 larval stages were identified by size and anatomical landmarks. All animals were imaged and fluorescence quantified as in Experimental Methods.
(TIFF)

Figure S10 Related to Figure 4. DAF-16 nuclear localization is unchanged in *pgrn-1(-)*, *ced-1(-)* or *ced-3(-)* mutants compared to wild type. Day 1 *daf-16(-)* adult animals expressing *P_daf-16_::daf-16::GFP* in an otherwise wild-type, *daf-2(e1370), pgrn-1(tm985)*, *ced-1(e1735)* or *ced-3(n717)* background were imaged on a Zeiss AxioImager and DAF-16::GFP was scored as either nuclear or diffuse. Images shown are representative of 10 to 15 animals observed for each genotype except for *ced-3(-)* for which three animals were observed. Top row shows two representative animals and bottom row displays close-ups of these animals.
(TIFF)

Figure S11 Lifespan analysis of *ced* mutants. Survival of N2 Control, *ced-3(n717), ced-1(e1735)* and *ced-2(e1752)* animals was plotted across time. Compared to control, *ced-3(-)* animals lived significantly longer (p = 0.0007, Mantel-Cox Test). N = 100 animals per strain.
(TIFF)

Figure S12 TNFR/neurotrophin receptor-related genes are not resistant to ER stress. (A) A *C. elegans* TRAF mutant, *trf-1(nr2010)*, was tested for stress resistance. (B) Two alleles of the neurotrophin receptor *trk-1* (*tm3985* and *tm4054*) were tested for ER stress resistance. Error bars represent standard deviation. N = 50 animals in triplicate per strain per condition. *p<0.05, **p<0.01, ***p<0.001.
(TIFF)

Table S1 Related to Figure 1. *pgrn-1* mutants are resistant to osmotic, thermal and unfolded protein stress. (A) Day-1 adult wild-type control animals and *pgrn-1(-)* mutants were exposed to osmotic stress with 600 mM NaCl for 24 hours and then scored for survival. Shown are mean survival ± SD and p value versus control (Student's t test). (B) Day-1 adult wild-type control animals and *pgrn-1(-)* mutants were exposed to thermal stress at 35°C for 8 hours and then scored for survival. Shown are mean survival ± SD and p value versus control (Student's t test). (C) Newly-laid wild-type control, *pgrn-1(tm985)* and *pgrn-1(-); pgrn-1*-rescue embryos were collected and placed onto plates with varying doses of tunicamycin. Three days later, those animals that had developed to L4 stage were counted. The fraction of animals that developed to L4 stage ± SD are shown. P value versus control and *pgrn-1* mutant are shown (ANOVA with Bonferroni post-tests). (D) Newly laid embryos from wild-type control, *pgrn-1(tm985)* and *pgrn-1(-); human PGRN*-rescue (2 independent lines) were collected and placed onto plates with varying doses of tunicamycin. Three days later, the number of animals that had developed to L4 stage was determined. The fraction of animals that developed to L4 stage ± SD are shown. P value versus control and *pgrn-1* mutant are shown (ANOVA with Bonferroni post-tests).
(DOCX)

Table S2 Related to Figure 2. c*ed-3(n717lf), ced-4(n162lf) and ced-9(n1950gf)* mutations inhibit programmed cell death and confer stress resistance. (A) ER stress resistance is correlated with *ced-3* allele strength. Newly laid embryos from wild-type control and *ced-3* mutant alleles with graded abilities to inhibit programmed cell death (*n717—strong, n1949—intermediate, n2436—intermediate and n2438—weak*) were collected and placed onto plates with varying doses of tunicamycin. Three days later, the number of animals that had developed to L4 stage was determined. The fraction of animals that developed to L4 stage ± SD is shown. P value versus control and *pgrn-1* mutant are shown (ANOVA with Bonferroni post-tests). (B) Day-1 adult wild-type control animals and *ced-3(-)* mutants were exposed to osmotic stress with 600 mM NaCl for 24 hours or thermal stress at 35°C for 8 hours and scored for survival. Shown are mean survival ± SD and p value versus control (Student's t test). (C) A gain-of-function mutation in *ced-9* increases stress resistance. Newly laid wild-type control, *pgrn-1(tm985)*, *ced-3(n717)*, *ced-9(n1950gf)*, *pgrn-1(-); ced-9(gf)* and *ced-3(n717); ced-9(gf)* embryos were collected and placed onto plates with varying doses of tunicamycin. Three days later, the number of animals that had developed to L4 stage was determined. The fraction of animals that developed to L4 stage ± SD are shown. P value versus control and *pgrn-1* mutant are shown (ANOVA with Bonferroni post-tests). (D) Newly laid wild-type control, *pgrn-1(tm985)*, *ced-4(n1162lf)* and *ced-4(n1162lf); ced-9(n2812lf)* embryos were collected and placed onto plates with varying doses of tunicamycin. Three days later, the number of animals that had developed to L4 stage was determined. The fraction of animals that developed to L4 stage ± SD are shown. P value versus control and *pgrn-1* mutant are shown (ANOVA with Bonferroni post-tests).
(DOCX)

Table S3 Related to Figure 3. Engulfment mutants are resistant to unfolded protein stress. (A) Newly laid wild-type control, *pgrn-1(tm985)*, *ced-1(e1735)*, *ced-6(n1813)* and *ced-7(n1892)* embryos were collected and placed onto plates with varying doses of tunicamycin. Three days later, the number of animals that had developed to L4 stage was determined. The fraction of animals that developed to L4 stage ± SD are shown. (B) Newly laid wild-type control, *pgrn-1(tm985)*, *ced-2(e1752)*, *ced-5(n1812)* and *ced-10(n3246)* embryos were collected and placed onto plates with varying doses of tunicamycin. Three days later, the number of animals that had developed to L4 stage was determined. The fraction of animals that developed to L4 stage ± SD are shown. (C) Day 1 adult wild-type control, *ced-1(e1735)*, *ced-6(n1813)*, *ced-7(n1892)*, *ced-2(e1752)* and *ced-5(n1812)* mutants were exposed to osmotic stress with 600 mM NaCl for 24 hours or thermal stress at 35°C for 10 hours and then scored for survival. Shown are mean survival ± SD and p value versus control (ANOVA with Tukey post-test). (D) Newly laid wild-type control, *pgrn-1(tm985)*, *abl-1(ok171)*, *abi-1(ok640)* and *abi-1(tm494)* embryos were collected and placed onto plates with varying doses of tunicamycin. Three days later, the number of animals that had developed to L4 stage was determined. The fraction of animals that developed to L4 stage ± SD are shown. P value versus control and *pgrn-1* mutant are shown (ANOVA with Bonferroni post-tests). (E) Newly laid wild-type control, *pgrn-1(tm985)*, *ced-1(n2091)*, *ced-1(n2091); abi-1(n1963)*, *ced-6(n2095)*, and *ced-6(n2095); abi-1(n1963)* embryos were collected and placed onto plates with varying doses of tunicamycin. Three days later, the number of animals that had developed to L4 stage was determined. The fraction of animals that developed to L4 stage ± SD are shown. P value versus control and *ced-1* or *ced-6* mutants are shown (ANOVA with Bonferroni post-tests). (F) Newly laid wild-type control, *pgrn-1(tm985)*, *unc-53(e404)*, and *unc-73(e936)* embryos were collected and placed onto plates with varying doses of tunicamycin. Three days later, the number of animals that had developed to L4 stage was determined. The fraction of animals that developed to L4 stage ± SD are shown. P value versus control and *pgrn-1* mutant are shown (ANOVA with Bonferroni post-tests). (G) Newly laid wild-type control, *pgrn-1(tm985)*, *pqn-41(ns294)*, and *pgrn-1(tm985); pqn-41(ns294)* embryos were collected and placed onto plates with varying doses of tunicamycin. Three days later, the number of animals that had developed to L4 stage was determined. The fraction of animals that developed to L4 stage ± SD are shown. P value versus control and *pgrn-1* mutant are shown (ANOVA with Bonferroni post-tests).
(DOCX)

Table S4 Related to Figure 4. *pgrn-1(-)* resistance to ER stress may be partially dependent on the UPR pathway, *daf-16* and *pmk-1*. (A) Newly laid embryos from wild-type control, *pgrn-1(tm985)*, *ire-1(ok799)* and *pgrn-1(-); ire-1(-)* mutants were collected and placed onto plates with varying doses of tunicamycin. Three days later, the number of animals that had developed to L4 stage was determined. The fraction of animals that developed to L4 stage ± SD is shown. P value versus control and *pgrn-1* mutant are shown (ANOVA with Bonferroni post-tests). (B) Newly laid embryos from wild-type control, *pgrn-1(tm985)*, *daf-2(e1370)*, *pgrn-1(-);daf-2(-)*, *daf-16(mu86)* and *pgrn-1(-); daf-16(-)* mutants were collected and placed onto plates with varying doses of tunicamycin. Three days later, the number of animals that had developed to L4 stage was determined. The fraction of animals that developed to L4 stage ± SD is shown. P value versus control and *pgrn-1* mutant are shown (ANOVA with Bonferroni post-tests). (C) Newly laid embryos from wild-type control, *pgrn-1(tm985)*, *daf-16(mu86); muIs113* and *pgrn-1(tm985); muIs113* mutants were collected and placed onto plates with varying doses of tunicamycin. Three days later, the number of animals that had developed to L4 stage was determined. The fraction of animals that developed to L4 stage ± SD is shown. P value versus control and *pgrn-1* mutant are shown (ANOVA with Bonferroni post-tests). (D) Newly laid embryos from wild-type control, *pgrn-1(tm985)*, *pmk-1(km25)* and *pgrn-1(-); pmk-1(-)* mutants were collected and placed onto plates with varying doses of tunicamycin. Three days later, the number of animals that had developed to L4 stage was determined. The fraction of animals that developed to L4 stage ± SD is shown. P value versus control and *pgrn-1* mutant are shown (ANOVA with Bonferroni post-tests).
(DOCX)

Table S5 Supplementary dataset of differentially expressed genes. Each worksheet contains lists of genes differentially expressed with FDR<0.05. Lists include log2 fold-changes, p-values, and FDR for each gene. Fold-changes are color coded to denote up- or down-regulation (up-regulated = red, down-regulated = green). Please see first tab for contents of other tabs. Tab E shows genes differentially expressed in *pgrn-1(tm985)*, *ced-3(n717)*, and *ced-1(e1735)* compared to N2E that are also regulated by *daf-16*. Hypergeometric distribution, p value<2×10^−9^.
(XLSX)
