# Supplementary figures and images for: A Shift to Organismal Stress Resistance in Programmed Cell Death Mutants
Source: PLoS Genet. 2013 Sep 19;9(9):e1003714. doi: 10.1371/journal.pgen.1003714 (PMC3778000; doi:10.1371/journal.pgen.1003714)

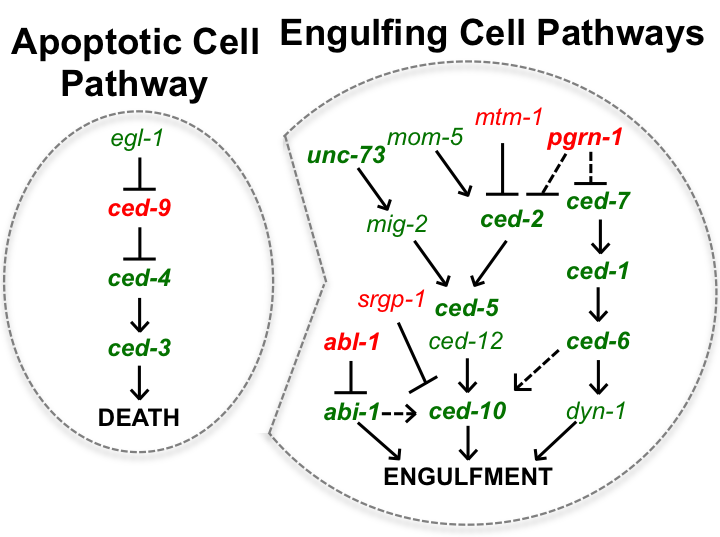

Supplement: Figure S1 — Genetic pathways that regulate programmed cell death in apoptotic and engulfing cells in C. elegans. Genes that normally promote cell death and engulfment are in green while those that normally inhibit them are shown in red. Genes tested in this study are in bold. (TIFF) [file pgen.1003714.s001.tiff]

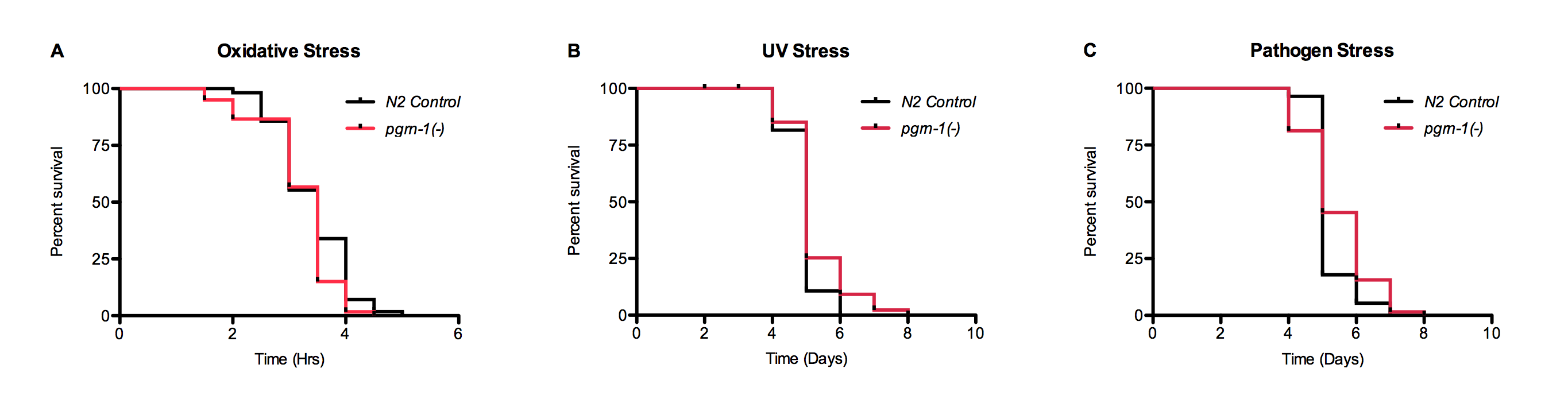

Supplement: Figure S2 — Related to Figure 1. A mutation in pgrn-1 does not confer oxidative, UV or pathogen stress resistance. Day 1 adult N2 or pgrn-1(tm985) animals were subjected to oxidative stress with 250 µM paraquat (A), genotoxic stress with 1200 J/m2 UV light (B) or pathogen stress by feeding P. aeruginosa (C). Worms were scored for survival at indicated times and analyses by log-rank Mantel-Cox test were performed. (A) p = 0.1, N>30 animals/strain. (B) p = 0.054, n>50 animals/strain. (C) p = 0.078, N>50 animals/strain. Results shown are representative of at least 2 experiments except in A, which was performed 3 times with p values of 0.0034, 0.01 and 0.125. (TIFF) [file pgen.1003714.s002.tiff]

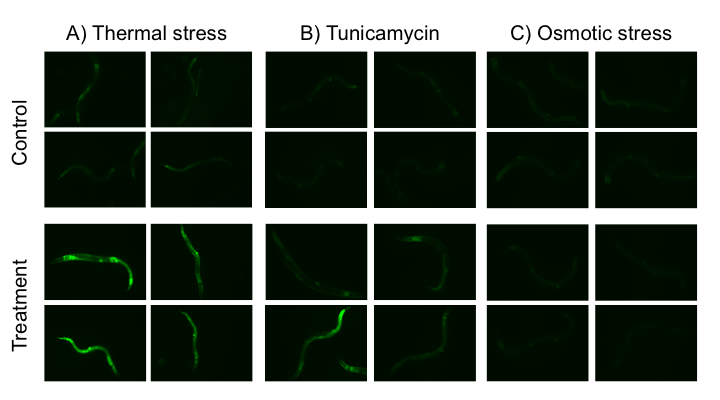

Supplement: Figure S3 — Related to Figure 1. Heat and tunicamycin stress increase HSP-4::GFP levels while osmotic stress does not. (A) Phsp-4::hsp-4::gfp animals were incubated at 20°C (Control treated) or 35°C (Heat stress treated) for 4 hours then imaged on a Zeiss AxioImager. (B) Phsp-4::hsp-4::gfp animals were incubated at 20°C on agarose plates containing 0 µg/mL (Control treated) or 5 µg/mL (ER stress treated) tunicamycin. Twenty-four hours later, they were imaged on a Zeiss AxioImager. (C) Phsp-4::hsp-4::gfp animals were incubated at 20°C on agarose plates containing 51 mM NaCl (Control treated) or 400 mM NaCl (Osmotic stress treated). After 24 hours, they were imaged on a Zeiss AxioImager. (TIFF) [file pgen.1003714.s003.tiff]

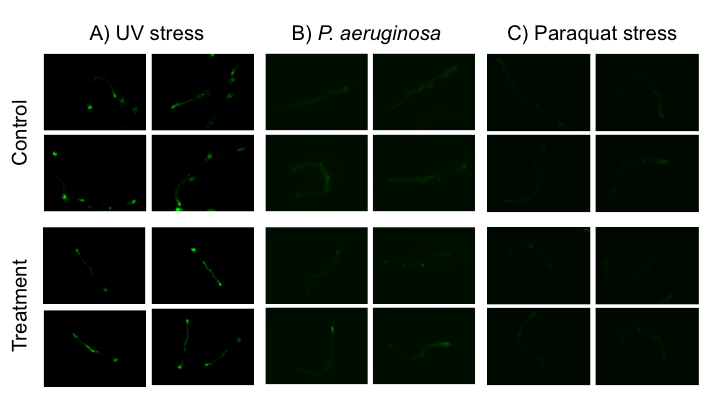

Supplement: Figure S4 — Related to Figure 1. UV light, paraquat and P. aeruginosa do not increase HSP-4::GFP levels. (A) Phsp-4::hsp-4::gfp animals were mock-treated (Control treated) or exposed to 1200 J/m2 of ultraviolet light (UV stress treated). After 5 hours at 20°C, the animals were imaged on a Zeiss AxioImager. (B) Phsp-4::hsp-4::gfp animals were placed onto agarose plates with either OP50 bacteria (Control treated) or P. aeruginosa PA14 (Pathogen treated) bacteria. After incubation for 24 hours at 20°C, animals were imaged on a Zeiss AxioImager. (C) Phsp-4::hsp-4::gfp animals were placed in M9 solution containing either vehicle alone (Control treated) or 200 mM paraquat (Oxidative stress treated) for 3 hours. The animals that were still alive at that time were imaged on a Zeiss AxioImager. (TIFF) [file pgen.1003714.s004.tiff]

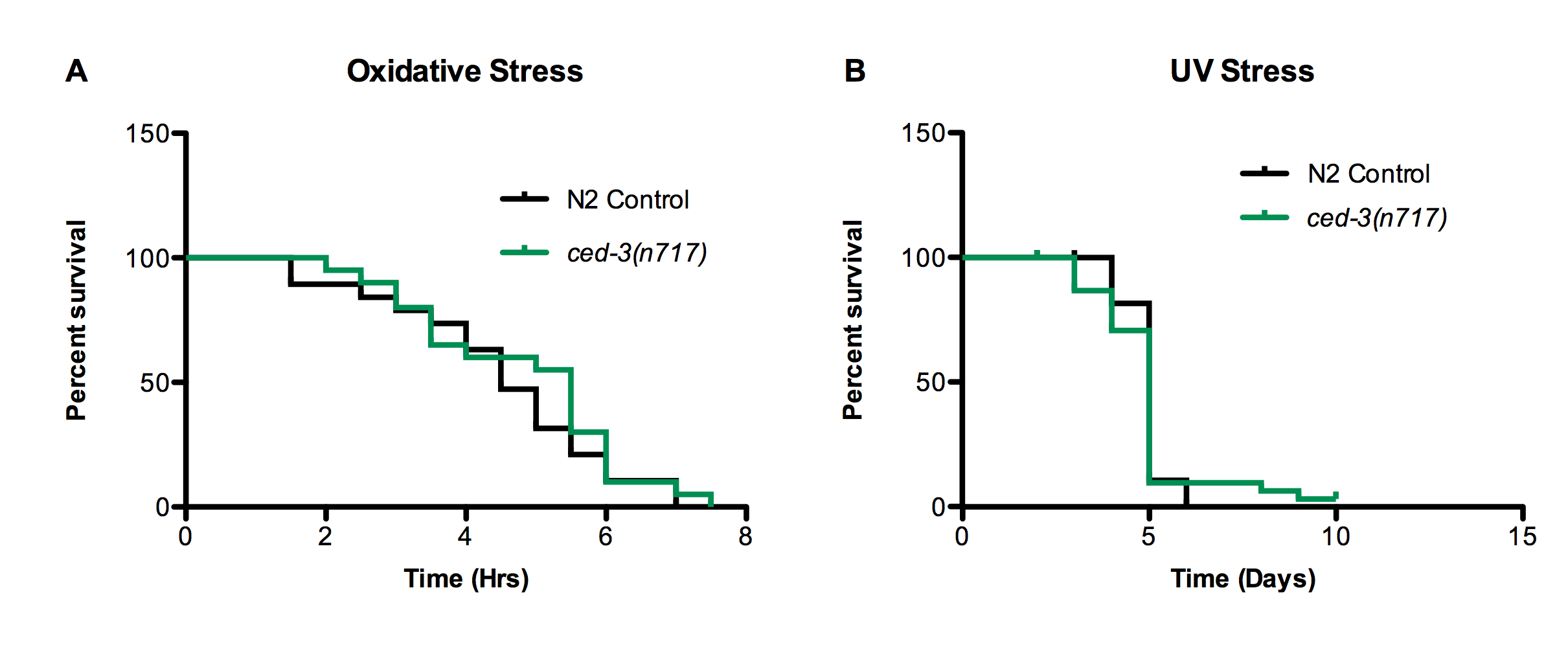

Supplement: Figure S5 — Related to Figure 2. A mutation in ced-3 does not confer oxidative or UV stress resistance. Day 1 adult N2 or ced-3(n717) animals were subjected to oxidative stress with 250 µM paraquat (A) or genotoxic stress with 1200 J/m2 UV light (B). Worms were scored for survival at indicated times and analyses by log-rank Mantel-Cox test were performed. (A) p>0.05, N = 20 animals/strain. (B) p>0.05, N>35 animals/strain. (TIFF) [file pgen.1003714.s005.tiff]

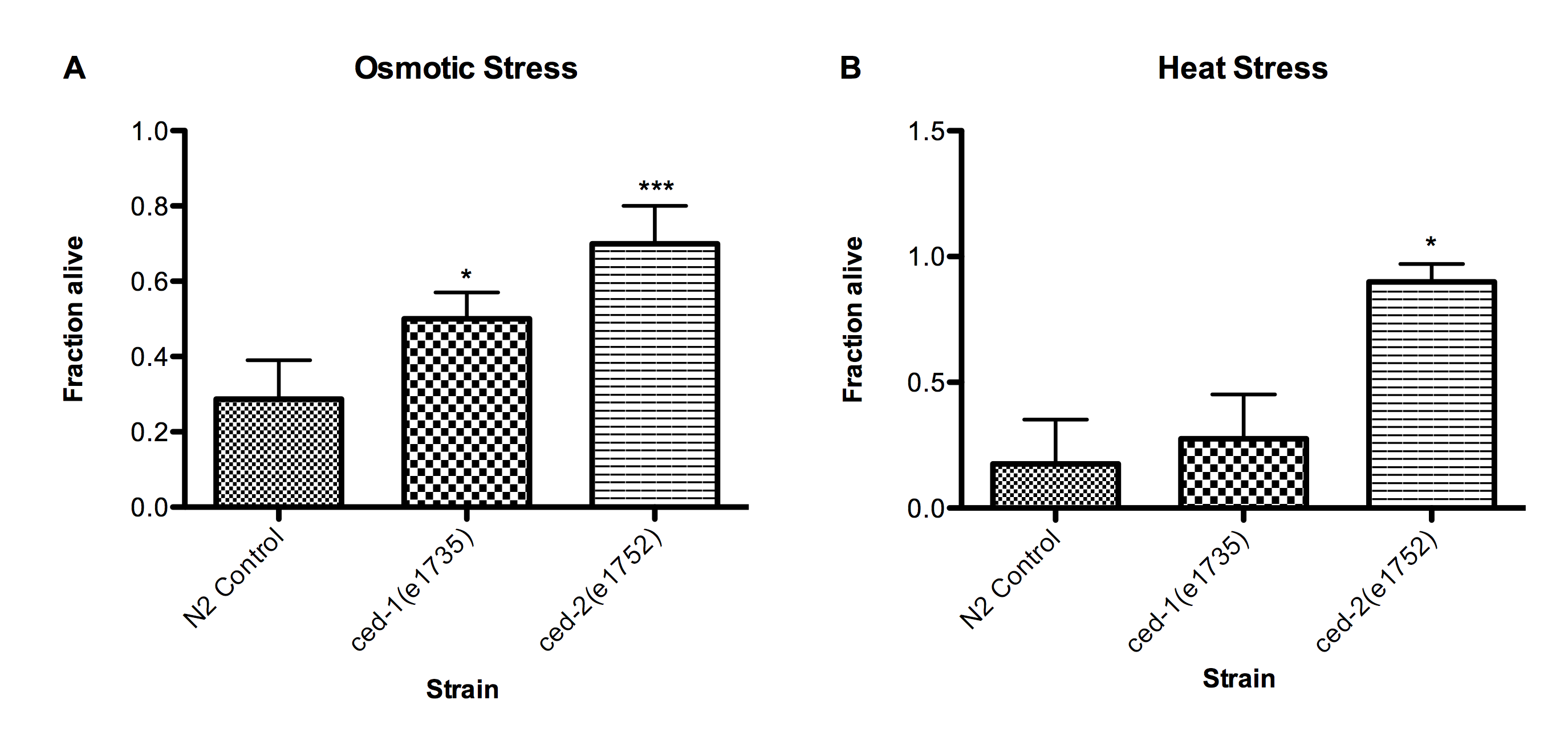

Supplement: Figure S6 — Related to Figure 3. Mutations in engulfment genes confer resistance to osmotic and heat stresses. Day 1 adult ced-1(e1735) and ced-2(e1752) animals were exposed to 600 mM NaCl for 24 hours (A) or thermal stress at 35°C for 8 hours (B) and scored for survival. Error bars represent standard deviation. Statistical comparisons here are to N2 control (Student's t test or ANOVA with Bonferroni post-tests). *p<0.05, ***p<0.001. For additional statistical data, see Supplemental Table S3C. (TIFF) [file pgen.1003714.s006.tiff]

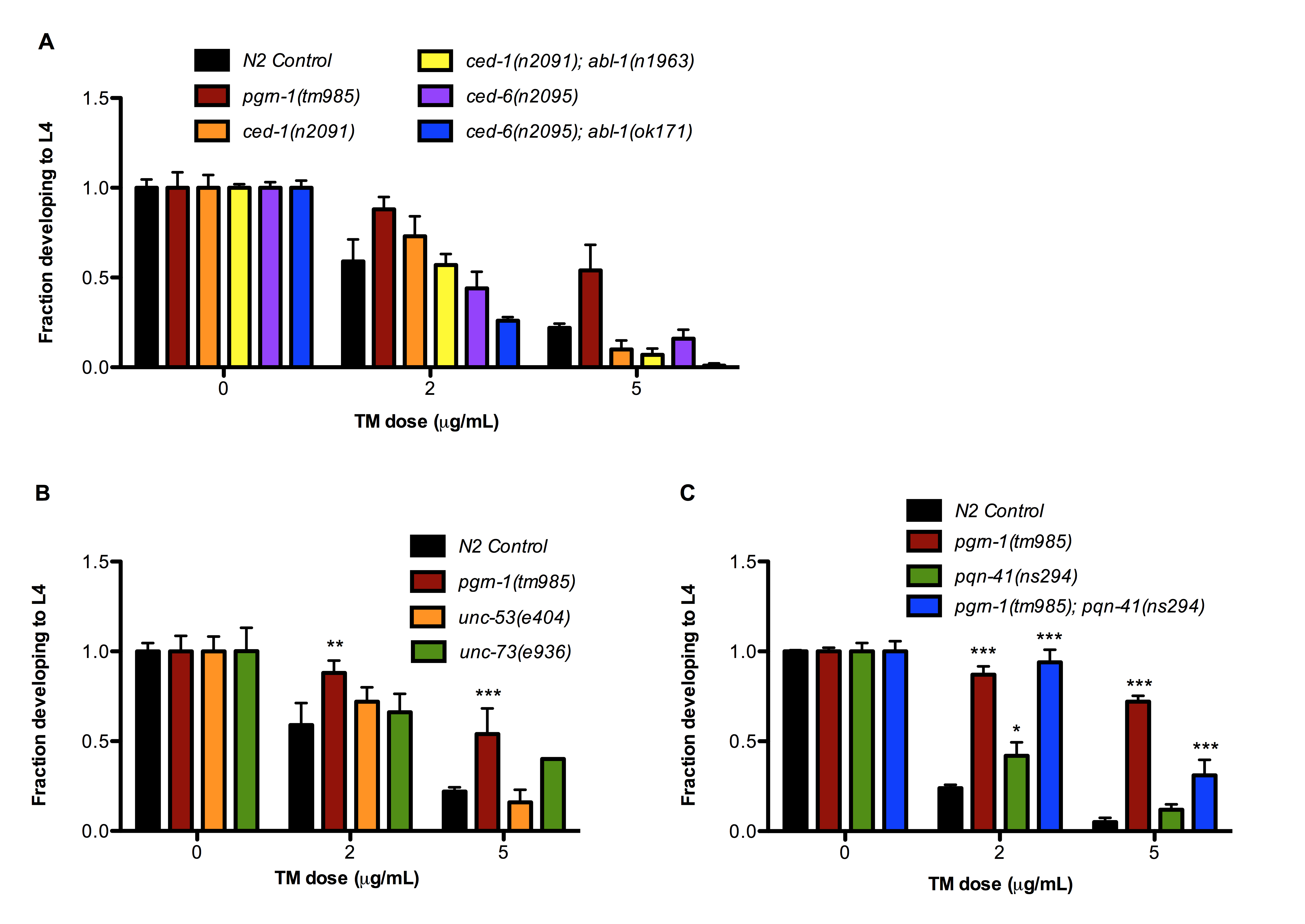

Supplement: Figure S7 — Related to Figure 3. ER stress resistance in related cell death mutants. A) Mutations in ced-1(e2091) and ced-6(n2095) with or without abi-1(n2091 or ok171) in the background were tested for ER stress resistance by tunicamycin (TM) treatment. (B) Mutations in unc-53(e404) and unc-73(e936) were tested for ER stress resistance by tunicamycin treatment. (C) Mutations in pqn-41(ns924) with and without pgrn-1(tm985) in the background were tested for ER stress resistance by tunicamycin treatment. Results shown are representative of at least 2 experiments except in the case of (C) which was performed once. Error bars represent standard deviation. Statistical comparisons are to N2 control (ANOVA with Bonferroni post-tests). *p<0.05, **p<0.01, ***p<0.001. For additional statistical data, see Supplemental Tables S3E–G. (TIFF) [file pgen.1003714.s007.tiff]

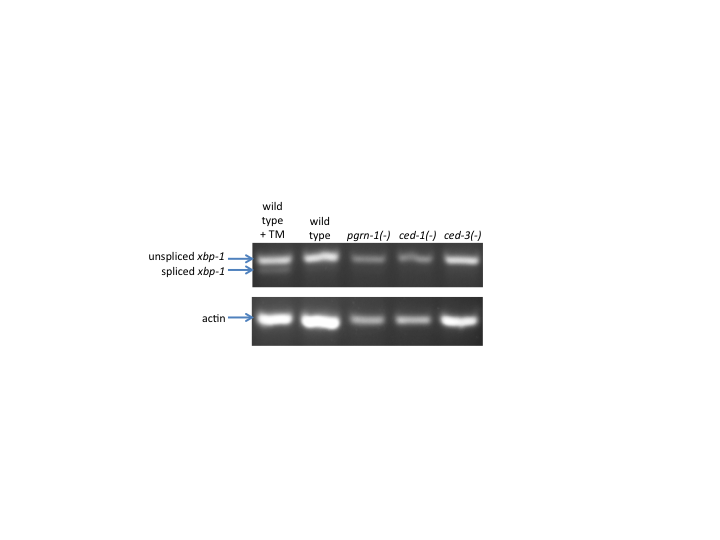

Supplement: Figure S8 — Related to Figure 4. Levels of spliced xbp-1 mRNA is unchanged in pgrn-1(-), ced-1(-) or ced-3(-) mutants compared to wild type. Representative RT-PCR of xbp-1 from RNA isolated from day 1 animals. Arrows point to the unspliced and spliced xbp-1 bands and the loading control actin band. + TM refers to treatment with 5 mg/mL tunicamycin. (TIFF) [file pgen.1003714.s008.tiff]

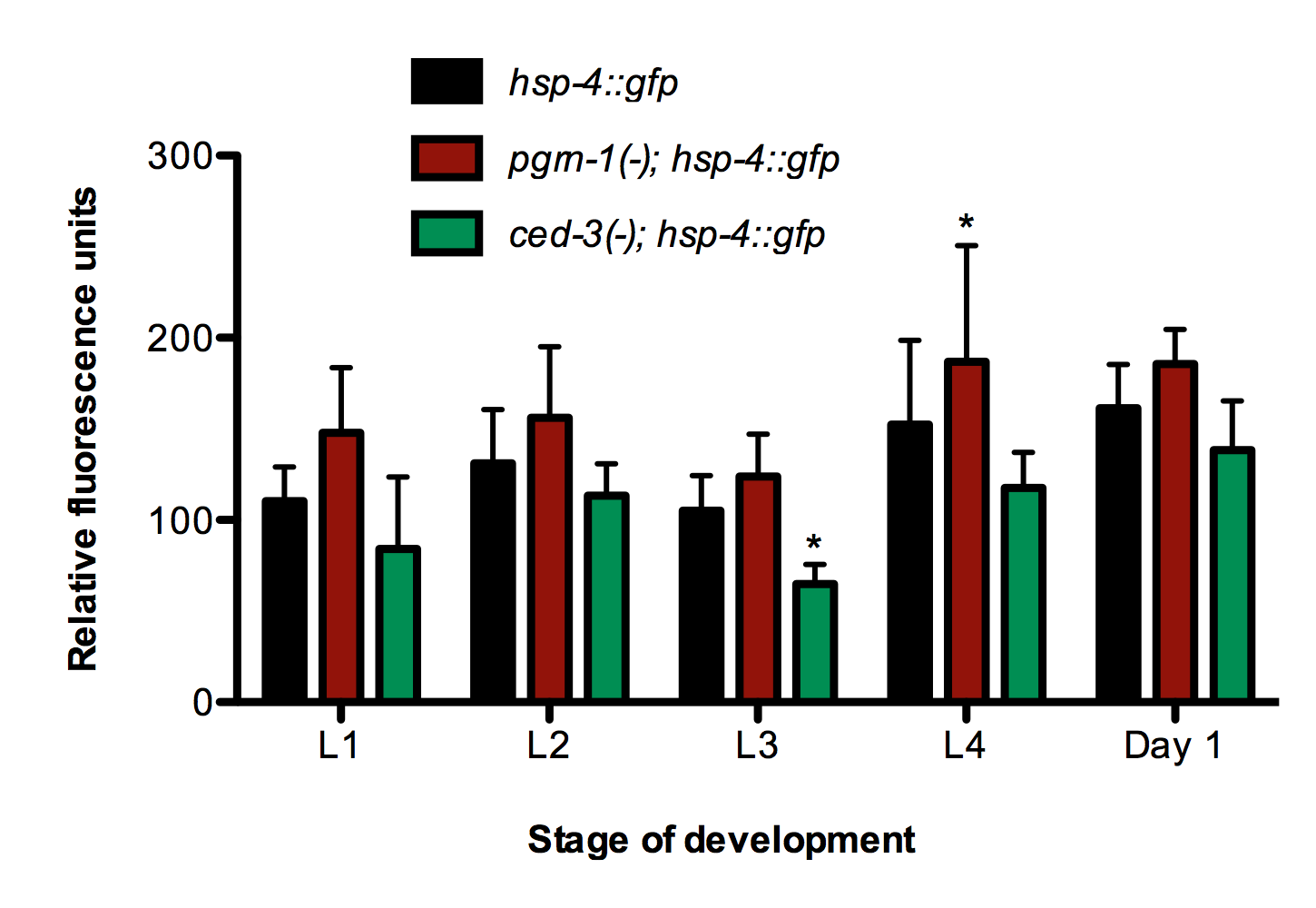

Supplement: Figure S9 — Related to Figure 4. HSP-4 does not mediate stress resistance of pgrn-1(-) or ced-3(-) mutants. Baseline levels of HSP-4::GFP in wild-type, pgrn-1(-) and ced-3(-) animals. Animals expressing Phsp-4::hsp-4::gfp in an otherwise wild-type, pgrn-1(tm985) or ced-3(n717) background were synchronized by washing adults and larvae away from eggs. L1 animals were collected from hatched embryos 1 hour later while L2, L3 and L4 larval stages were identified by size and anatomical landmarks. All animals were imaged and fluorescence quantified as in Experimental Methods. (TIFF) [file pgen.1003714.s009.tiff]

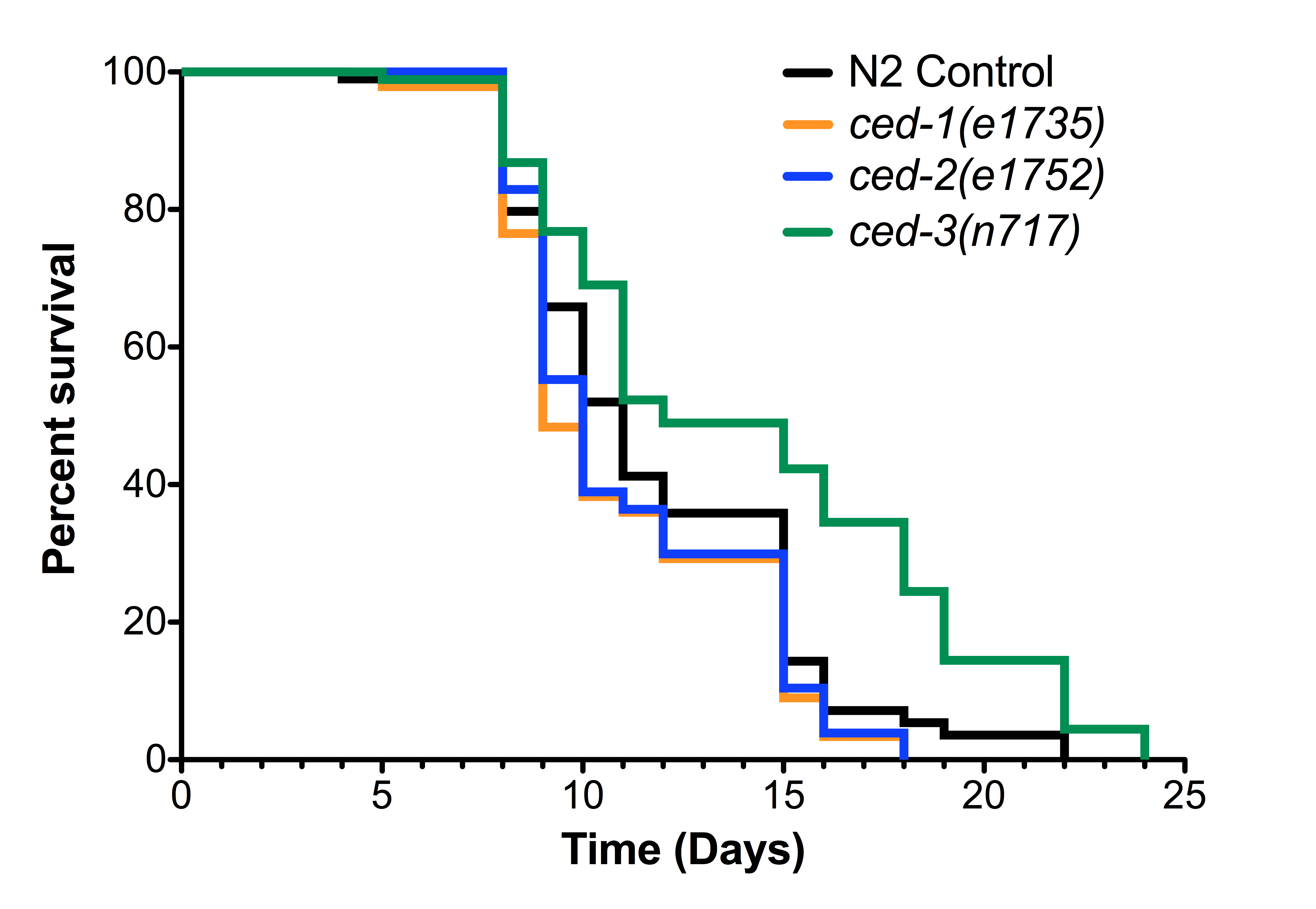

Supplement: Figure S11 — Lifespan analysis of ced mutants. Survival of N2 Control, ced-3(n717), ced-1(e1735) and ced-2(e1752) animals was plotted across time. Compared to control, ced-3(-) animals lived significantly longer (p = 0.0007, Mantel-Cox Test). N = 100 animals per strain. (TIFF) [file pgen.1003714.s011.tiff]

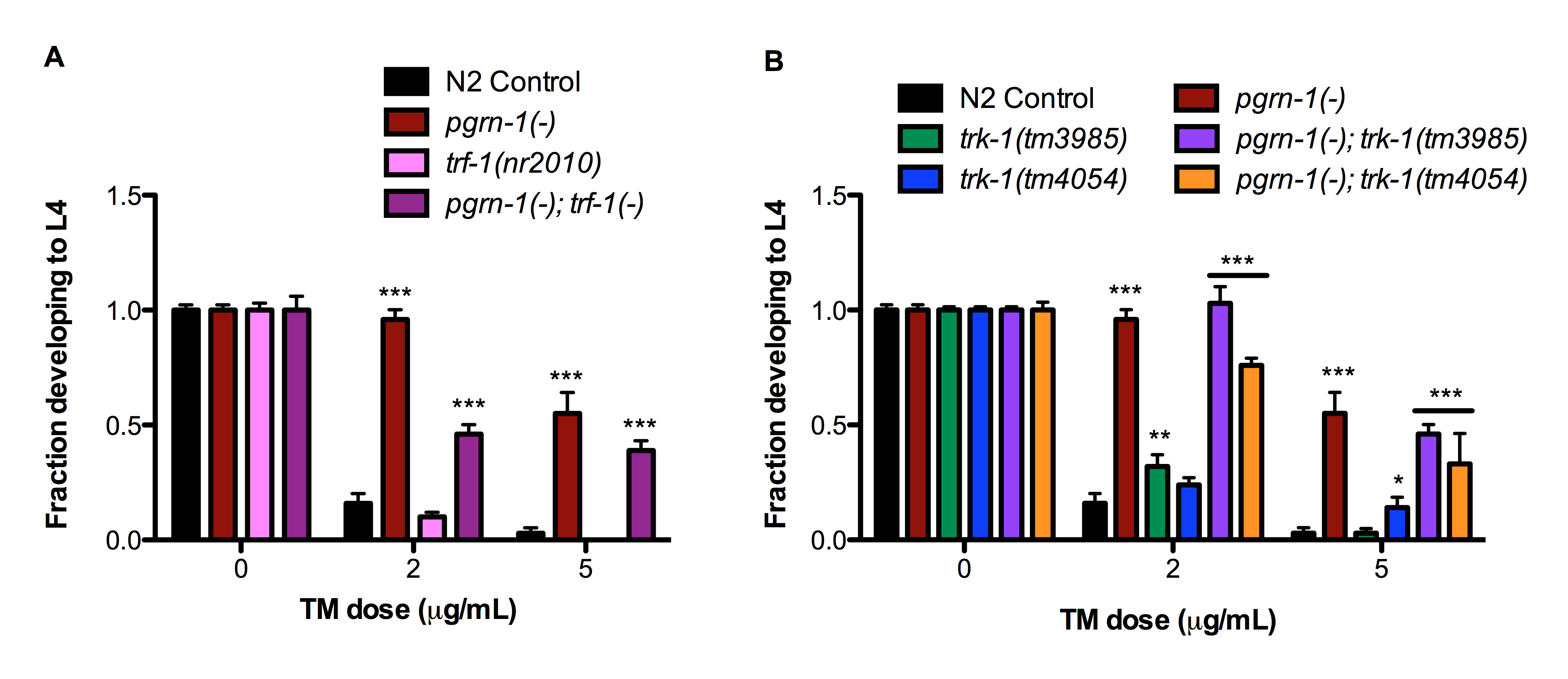

Supplement: Figure S12 — TNFR/neurotrophin receptor-related genes are not resistant to ER stress. (A) A C. elegans TRAF mutant, trf-1(nr2010), was tested for stress resistance. (B) Two alleles of the neurotrophin receptor trk-1 (tm3985 and tm4054) were tested for ER stress resistance. Error bars represent standard deviation. N = 50 animals in triplicate per strain per condition. *p<0.05, **p<0.01, ***p<0.001. (TIFF) [file pgen.1003714.s012.tiff]
