# Supplementary material for: A Shift to Organismal Stress Resistance in Programmed Cell Death Mutants
Source: PLoS Genet. 2013 Sep 19;9(9):e1003714. doi: 10.1371/journal.pgen.1003714 (PMC3778000; doi:10.1371/journal.pgen.1003714)
Supplement: Table S1 — Related to Figure 1. pgrn-1 mutants are resistant to osmotic, thermal and unfolded protein stress. (A) Day-1 adult wild-type control animals and pgrn-1(-) mutants were exposed to osmotic stress with 600 mM NaCl for 24 hours and then scored for survival. Shown are mean survival ± SD and p value versus control (Student's t test). (B) Day-1 adult wild-type control animals and pgrn-1(-) mutants were exposed to thermal stress at 35°C for 8 hours and then scored for survival. Shown are mean survival ± SD and p value versus control (Student's t test). (C) Newly-laid wild-type control, pgrn-1(tm985) and pgrn-1(-); pgrn-1-rescue embryos were collected and placed onto plates with varying doses of tunicamycin. Three days later, those animals that had developed to L4 stage were counted. The fraction of animals that developed to L4 stage ± SD are shown. P value versus control and pgrn-1 mutant are shown (ANOVA with Bonferroni post-tests). (D) Newly laid embryos from wild-type control, pgrn-1(tm985) and pgrn-1(-); human PGRN-rescue (2 independent lines) were collected and placed onto plates with varying doses of tunicamycin. Three days later, the number of animals that had developed to L4 stage was determined. The fraction of animals that developed to L4 stage ± SD are shown. P value versus control and pgrn-1 mutant are shown (ANOVA with Bonferroni post-tests). (DOCX) [file pgen.1003714.s013.docx]

**Supplemental Table S1** *Indicates experiment shown in Figures.

| **Table S1A. Effect of *pgrn-1(tm985)* mutation on osmotic stress resistance** | | | | | |
| --- | --- | --- | --- | --- | --- |
| **Treatment** | **Repeat #** | **Genotype** | **Mean survival ± SD** | **N** | **P vs.**  **control** |
| Osmotic stress | 1* | Control | 0.22 ± 0.08 | N = 60 | -- |
|  |  | *pgrn-1* | 0.42 ± 0.10 | N = 60 | 0.03 |
|  | 2 | Control | 0.41 ± 0.08 | N = 60 | -- |
|  |  | *pgrn-1* | 0.52 ± 0.06 | N = 60 | 0.12 |
|  | 3 | Control | 0.03 ± 0.03 | N = 60 |  |
|  |  | *pgrn-1* | 0.34 ± 0.04 | N = 60 | 0.0004 |

| **Table S1B. Effect of *pgrn-1(tm985)* mutation on thermal stress resistance** | | | | | |
| --- | --- | --- | --- | --- | --- |
| **Treatment** | **Repeat #** | **Genotype** | **Mean survival ± SD** | **N** | **P vs.**  **control** |
| Thermotolerance | 1* | Control | 0.25 ± 0.14 | N = 60 | -- |
|  |  | *pgrn-1* | 0.57 ± 0.09 | N = 40 | 0.03 |
|  | 2 | Control | 0.58 ± 0.13 | N = 61 | -- |
|  |  | *pgrn-1* | 0.90 ± 0.00 | N = 60 | 0.04 |
|  | 3 | Control | 0.46 ± 0.13 | N = 61 | -- |
|  |  | *pgrn-1* | 0.93 ± 0.03 | N = 60 | 0.004 |

| **Table S1C. Effect of *pgrn-1(tm985)* mutation and rescue on ER stress resistance in developing worms** | | | | | | | |
| --- | --- | --- | --- | --- | --- | --- | --- |
| **Treatment** | **Repeat #** | **Tunicamycin**  **(µg/mL)** | **Genotype** | **Fraction developing to L4 ± SD** | **N** | **P vs.**  **control** | **P vs. *pgrn-1*** |
| ER stress | 1* | 0 | Control | 1.00 ± 0.02 | N = 205 | -- | -- |
|  |  |  | *pgrn-1* | 1.00 ± 0.02 | N = 259 | n.s. | -- |
|  |  |  | *pgrn-1* rescue | 1.00 ± 0.04 | N = 154 | n.s. | n.s. |
|  |  | 1 | Control | 0.27 ± 0.14 | N = 197 | -- | -- |
|  |  |  | *pgrn-1* | 0.80 ± 0.01 | N = 200 | P < 0.001 | -- |
|  |  |  | *pgrn-1* rescue | 0.36 ± 0.16 | N = 138 | n.s. | P < 0.001 |
|  |  | 2 | Control | 0.04 ± 0.02 | N = 171 | -- | -- |
|  |  |  | *pgrn-1* | 0.55 ± 0.02 | N = 228 | P < 0.001 | -- |
|  |  |  | *pgrn-1* rescue | 0.02 ± 0.02 | N = 144 | n.s. | P < 0.001 |
|  |  | 5 | Control | 0.05 ± 0.02 | N = 192 | -- | -- |
|  |  |  | *pgrn-1* | 0.57 ± 0.07 | N = 205 | P < 0.001 | -- |
|  |  |  | *pgrn-1* rescue | 0.03 ± 0.04 | N = 156 | n.s. | P < 0.001 |
|  | 2 | 0 | Control | 1.00 ± 0.03 | N = 250 | -- | -- |
|  |  |  | *pgrn-1* | 1.00 ± 0.04 | N = 186 | n.s. | -- |
|  |  |  | *pgrn-1* rescue | 1.00 ± 0.12 | N = 177 | n.s. | n.s. |
|  |  | 1 | Control | 0.05 ± 0.05 | N = 176 | -- | -- |
|  |  |  | *pgrn-1* | 0.70 ± 0.20 | N = 184 | P < 0.001 | -- |
|  |  |  | *pgrn-1* rescue | 0.59 ± 0.09 | N = 159 | P < 0.001 | n.s. |
|  |  | 2 | Control | 0.00 ± 0.00 | N = 194 | -- | -- |
|  |  |  | *pgrn-1* | 0.81 ± 0.18 | N = 160 | P < 0.001 | -- |
|  |  |  | *pgrn-1* rescue | 0.36 ± 0.04 | N = 156 | P < 0.001 | P < 0.001 |
|  |  | 5 | Control | 0.02 ± 0.00 | N = 191 | -- | -- |
|  |  |  | *pgrn-1* | 0.51 ± 0.06 | N = 194 | P < 0.001 | -- |
|  |  |  | *pgrn-1* rescue | 0.07 ± 0.00 | N = 166 | n.s. | P < 0.001 |

| **Table S1D. Human progranulin partially rescues *pgrn-1* mutant ER stress resistance** | | | | | | | |
| --- | --- | --- | --- | --- | --- | --- | --- |
| **Treatment** | **Repeat #** | **Tunicamycin**  **(µg/mL)** | **Genotype** | **Fraction developing to L4 ± SD** | **N** | **P vs.**  **control** | **P vs. *pgrn-1*** |
| ER stress | 1* | 0 | Control | 1.00 ± 0.01 | N = 150 | -- | -- |
|  |  |  | *pgrn-1* | 1.00 ± 0.06 | N = 150 | n.s. | -- |
|  |  |  | *huPGRN1* | 1.00 ± 0.02 | N = 150 | n.s. | n.s. |
|  |  |  | *huPGRN2* | 1.00 ± 0.06 | N = 150 | n.s. | n.s. |
|  |  | 1 | Control | 0.10 ± 0.05 | N = 150 | -- | -- |
|  |  |  | *pgrn-1* | 0.82 ± 0.05 | N = 150 | P < 0.001 | -- |
|  |  |  | *huPGRN1* | 0.80 ± 0.06 | N = 150 | P < 0.001 | n.s. |
|  |  |  | *huPGRN2* | 0.87 ± 0.16 | N = 150 | P < 0.001 | n.s. |
|  |  | 5 | Control | 0.08 ± 0.03 | N = 150 | -- | -- |
|  |  |  | *pgrn-1* | 0.71 ± 0.05 | N = 150 | P < 0.001 | -- |
|  |  |  | *huPGRN1* | 0.26 ± 0.11 | N = 150 | n.s. | P < 0.001 |
|  |  |  | *huPGRN2* | 0.21 ± 0.08 | N = 150 | n.s. | P < 0.001 |
|  | 2 | 0 | Control | 1.00 ± 0.06 | N = 150 | -- | -- |
|  |  |  | *pgrn-1* | 1.00 ± 0.02 | N = 150 | n.s. | -- |
|  |  |  | *huPGRN1* | 1.00 ± 0.03 | N = 150 | n.s. | n.s. |
|  |  |  | *huPGRN2* | 1.00 ± 0.05 | N = 150 | n.s. | n.s. |
|  |  | 1 | Control | 0.09 ± 0.08 | N = 150 | -- | -- |
|  |  |  | *pgrn-1* | 0.84 ± 0.03 | N = 150 | P < 0.001 | -- |
|  |  |  | *huPGRN1* | 0.82 ± 0.02 | N = 150 | P < 0.001 | n.s. |
|  |  |  | *huPGRN2* | 0.94 ± 0.10 | N = 150 | P < 0.001 | n.s. |
|  |  | 5 | Control | 0.05 ± 0.03 | N = 150 | -- | -- |
|  |  |  | *pgrn-1* | 0.66 ± 0.13 | N = 150 | P < 0.001 | -- |
|  |  |  | *huPGRN1* | 0.34 ± 0.02 | N = 150 | P < 0.001 | P < 0.001 |
|  |  |  | *huPGRN2* | 0.33 ± 0.08 | N = 150 | P < 0.001 | P < 0.001 |
