# Supplementary material for: A Shift to Organismal Stress Resistance in Programmed Cell Death Mutants
Source: PLoS Genet. 2013 Sep 19;9(9):e1003714. doi: 10.1371/journal.pgen.1003714 (PMC3778000; doi:10.1371/journal.pgen.1003714)
Supplement: Table S2 — Related to Figure 2. ced-3(n717lf), ced-4(n162lf) and ced-9(n1950gf) mutations inhibit programmed cell death and confer stress resistance. (A) ER stress resistance is correlated with ced-3 allele strength. Newly laid embryos from wild-type control and ced-3 mutant alleles with graded abilities to inhibit programmed cell death (n717—strong, n1949—intermediate, n2436—intermediate and n2438—weak) were collected and placed onto plates with varying doses of tunicamycin. Three days later, the number of animals that had developed to L4 stage was determined. The fraction of animals that developed to L4 stage ± SD is shown. P value versus control and pgrn-1 mutant are shown (ANOVA with Bonferroni post-tests). (B) Day-1 adult wild-type control animals and ced-3(-) mutants were exposed to osmotic stress with 600 mM NaCl for 24 hours or thermal stress at 35°C for 8 hours and scored for survival. Shown are mean survival ± SD and p value versus control (Student's t test). (C) A gain-of-function mutation in ced-9 increases stress resistance. Newly laid wild-type control, pgrn-1(tm985), ced-3(n717), ced-9(n1950gf), pgrn-1(-); ced-9(gf) and ced-3(n717); ced-9(gf) embryos were collected and placed onto plates with varying doses of tunicamycin. Three days later, the number of animals that had developed to L4 stage was determined. The fraction of animals that developed to L4 stage ± SD are shown. P value versus control and pgrn-1 mutant are shown (ANOVA with Bonferroni post-tests). (D) Newly laid wild-type control, pgrn-1(tm985), ced-4(n1162lf) and ced-4(n1162lf); ced-9(n2812lf) embryos were collected and placed onto plates with varying doses of tunicamycin. Three days later, the number of animals that had developed to L4 stage was determined. The fraction of animals that developed to L4 stage ± SD are shown. P value versus control and pgrn-1 mutant are shown (ANOVA with Bonferroni post-tests). (DOCX) [file pgen.1003714.s014.docx]

**Supplemental Table S2** *Indicates experiment shown in Figures.

| **Table S2A. Effect of *ced-3* allele strength on ER stress resistance** | | | | | | | |
| --- | --- | --- | --- | --- | --- | --- | --- |
| ***ced-3***  **allele** | **Repeat #** | **Tunicamycin**  **(µg/mL)** | **Genotype** | **Fraction developing to L4 ± SD** | **N** | **P vs.**  **control** | **P vs. *pgrn-1*** |
| *n717* | 1* | 0 | Control | 1.00 ± 0.02 | N = 150 | -- | -- |
|  |  |  | *pgrn-1* | 1.00 ± 0.04 | N = 150 | n.s. | -- |
|  |  |  | *ced-3* | 1.00 ± 0.07 | N = 150 | n.s. | n.s. |
|  |  |  | *pgrn-1; ced-3* | 1.00 ± 0.05 | N = 150 | n.s. | n.s. |
|  |  | 1 | Control | 0.08 ± 0.04 | N = 150 | -- | -- |
|  |  |  | *pgrn-1* | 0.72 ± 0.04 | N = 150 | P < 0.001 | -- |
|  |  |  | *ced-3* | 0.71 ± 0.10 | N = 150 | P < 0.001 | n.s. |
|  |  |  | *pgrn-1; ced-3* | 0.69 ± 0.07 | N = 150 | P < 0.001 | n.s. |
|  |  | 5 | Control | 0.14 ± 0.01 | N = 150 | -- | -- |
|  |  |  | *pgrn-1* | 0.47 ± 0.02 | N = 150 | P < 0.001 | -- |
|  |  |  | *ced-3* | 0.47 ± 0.02 | N = 150 | P < 0.001 | n.s. |
|  |  |  | *pgrn-1; ced-3* | 0.51 ± 0.06 | N = 150 | P < 0.001 | n.s. |
|  | 2 | 0 | Control | 1.00 ± 0.00 | N = 150 | -- | -- |
|  |  |  | *pgrn-1* | 1.00 ± 0.07 | N = 150 | n.s. | -- |
|  |  |  | *ced-3* | 1.00 ± 0.02 | N = 150 | n.s. | n.s. |
|  |  |  | *pgrn-1; ced-3* | 1.00 ± 0.07 | N = 150 | n.s. | n.s. |
|  |  | 1 | Control | 019 ± 0.05 | N = 150 | -- | -- |
|  |  |  | *pgrn-1* | 0.80 ± 0.05 | N = 150 | P < 0.001 | -- |
|  |  |  | *ced-3* | 0.93 ± 0.14 | N = 150 | P < 0.001 | n.s. |
|  |  |  | *pgrn-1; ced-3* | 0.88 ± 0.04 | N = 150 | P < 0.001 | n.s. |
|  |  | 5 | Control | 0.19 ± 0.07 | N = 150 | -- | -- |
|  |  |  | *pgrn-1* | 0.56 ± 0.05 | N = 150 | P < 0.001 | -- |
|  |  |  | *ced-3* | 0.53 ± 0.18 | N = 150 | P < 0.001 | n.s. |
|  |  |  | *pgrn-1; ced-3* | 0.51 ± 0.03 | N = 150 | P < 0.001 | n.s. |
|  | 3 | 0 | Control | 1.00 ± 0.10 | N = 150 | -- | -- |
|  |  |  | *pgrn-1* | 1.00 ± 0.04 | N = 150 | n.s. | -- |
|  |  |  | *ced-3* | 1.00 ± 0.08 | N = 150 | n.s. | n.s. |
|  |  | 1 | Control | 0.15 ± 0.05 | N = 150 | -- | -- |
|  |  |  | *pgrn-1* | 0.69 ± 0.15 | N = 150 | P < 0.001 | -- |
|  |  |  | *ced-3* | 0.70 ± 0.12 | N = 150 | P < 0.001 | n.s. |
|  |  | 2 | Control | 0.03 ± 0.02 | N = 150 | -- | -- |
|  |  |  | *pgrn-1* | 0.17 ± 0.06 | N = 150 | P < 0.001 | -- |
|  |  |  | *ced-3* | 0.35 ± 0.09 | N = 150 | P < 0.001 | n.s. |
|  |  |  |  |  |  |  |  |
| Allelic series | 1* | 0 | Control | 1.00 ± 0.01 | N = 100 | -- | -- |
|  |  |  | *ced-3(n717)* | 1.00 ± 0.03 | N = 150 | n.s. | -- |
|  |  |  | *ced-3(n1949)* | 1.00 ± 0.10 | N = 150 | n.s. | -- |
|  |  |  | *ced-3(n2436)* | 1.00 ± 0.03 | N = 150 | n.s. | -- |
|  |  |  | *ced-3(n2438)* | 1.00 ± 0.02 | N = 150 | n.s. |  |
|  |  | 2 | Control | 0.01 ± 0.01 | N = 150 | -- | -- |
|  |  |  | *ced-3(n717)* | 0.46 ± 0.02 | N = 150 | P < 0.001 | -- |
|  |  |  | *ced-3(n1949)* | 0.45 ± 0.07 | N = 150 | P < 0.001 | -- |
|  |  |  | *ced-3(n2436)* | 0.39 ± 0.05 | N = 150 | P < 0.001 | -- |
|  |  |  | *ced-3(n2438)* | 0.12 ± 0.05 | N = 150 | P < 0.01 |  |
|  |  | 5 | Control | 0.00 ± 0.00 | N = 150 | -- | -- |
|  |  |  | *ced-3(n717)* | 0.13 ± 0.03 | N = 150 | P < 0.01 | -- |
|  |  |  | *ced-3(n1949)* | 0.09 ± 0.04 | N = 150 | n.s. | -- |
|  |  |  | *ced-3(n2436)* | 0.00 ± 0.00 | N = 150 | n.s. | -- |
|  |  |  | *ced-3(n2438)* | 0.00 ± 0.00 | N = 150 | n.s. |  |
|  | 2 | 0 | Control | 1.00 ± 0.01 | N = 150 | -- | -- |
|  |  |  | *ced-3(n717)* | 1.00 ± 0.03 | N = 150 | n.s. | -- |
|  |  |  | *ced-3(n1949)* | 1.00 ± 0.06 | N = 150 | n.s. | -- |
|  |  |  | *ced-3(n2436)* | 1.00 ± 0.06 | N = 150 | n.s. | -- |
|  |  |  | *ced-3(n2438)* | 1.00 ± 0.03 | N = 150 | n.s. |  |
|  |  | 5 | Control | 0.01 ± 0.14 | N = 150 | -- | -- |
|  |  |  | *ced-3(n717)* | 0.14 ± 0.03 | N = 150 | P < 0.01 | -- |
|  |  |  | *ced-3(n1949)* | 0.07 ± 0.02 | N = 150 | n.s. | -- |
|  |  |  | *ced-3(n2436)* | 0.03 ± 0.01 | N = 150 | n.s. | -- |
|  |  |  | *ced-3(n2438)* | 0.00 ± 0.00 | N = 150 | n.s. |  |

| **Table S2B. Effect of *ced-3(n717)* mutation on heat and osmotic stress resistance** | | | | | |
| --- | --- | --- | --- | --- | --- |
| **Treatment** | **Repeat #** | **Genotype** | **Mean survival ± SD** | **N** | **P vs.**  **control** |
| Osmotic Stress | 1* | Control | 0.65 ± 0.06 | N = 17 | -- |
|  |  | *ced-3* | 0.92 ± 0.005 | N = 23 | 0.04 |
|  | 2 | Control | 0.29 ± 0.10 | N = 80 | -- |
|  |  | *ced-3* | 0.08 ± 0.05 | N = 80 | P < 0.005 |
|  |  |  |  |  |  |
| Thermotolerance | 1* | Control | 0.22 ± 0.07 | N = 60 | -- |
|  |  | *ced-3* | 0.5 ± 0.1 | N = 60 | 0.08 |
|  | 2* | Control | 0.33 ± 0.02 | N = 60 | -- |
|  |  | *ced-3* | 0.48 ± 0.07 | N = 60 | 0.09 |
|  | 3* | Control | 0.03 ± 0.016 | N = 60 | -- |
|  |  | *ced-3* | 0.18 ± 0.09 | N = 60 | 0.17 |

| **Table S2C. ER stress resistance of *ced-9(n1950gf)* mutants** | | | | | | | |
| --- | --- | --- | --- | --- | --- | --- | --- |
| **Repeat #** | **Tunicamycin**  **(µg/mL)** | **Genotype** | **Fraction developing to L4 ± SD** | **N** | **P vs.**  **control** | **P vs.  *pgrn-1*** | **P vs. *ced-3*** |
| 1* | 0 | Control | 1.00 ± 0.00 | N = 100 | -- | -- | -- |
|  |  | *pgrn-1* | 1.00 ± 0.00 | N = 100 | n.s. | -- | -- |
|  |  | *ced-3(n717)* | 1.00 ± 0.09 | N = 100 | n.s. | n.s. | -- |
|  |  | *ced-9(n1950)* | 1.00 ± 0.06 | N = 100 | n.s. | n.s. | n.s. |
|  |  | *pgrn-1; ced-9* | 1.00 ± 0.01 | N = 100 | n.s. | n.s. | n.s. |
|  |  | *ced-9; ced-3* | 1.00 ± 0.03 | N = 150 | n.s. | n.s. | n.s. |
|  | 2.5 | Control | 0.03 ± 0.01 | N = 100 | -- | -- | -- |
|  |  | *pgrn-1* | 0.31 ± 0.04 | N = 100 | P < 0.001 | -- | -- |
|  |  | *ced-3(n717)* | 0.53 ± 0.00 | N = 100 | P < 0.001 | P < 0.001 | -- |
|  |  | *ced-9(n1950)* | 0.66 ± 0.02 | N = 150 | P < 0.001 | P < 0.001 | P < 0.05 |
|  |  | *pgrn-1; ced-9* | 0.89 ± 0.03 | N = 150 | P < 0.001 | P < 0.001 | P < 0.001 |
|  |  | *ced-9; ced-3* | 0.64 ± 0.04 | N = 100 | P < 0.001 | P < 0.001 | n.s. |
|  | 5 | Control | 0.00 ± 0.00 | N = 100 | **--** | -- | -- |
|  |  | *pgrn-1* | 0.48 ± 0.01 | N = 100 | P < 0.001 | -- | -- |
|  |  | *ced-3(n717)* | 0.58 ± 0.03 | N = 100 | P < 0.001 | n.s | -- |
|  |  | *ced-9(n1950)* | 0.63 ± 0.01 | N = 100 | P < 0.001 | P < 0.05 | n.s. |
|  |  | *pgrn-1; ced-9* | 0.60 ± 0.11 | N = 150 | P < 0.001 | n.s. | n.s. |
|  |  | *ced-9; ced-3* | 0.51 ± 0.09 | N = 150 | P < 0.001 | n.s. | n.s. |
| 2 | 0 | Control | 1.00 ± 0.00 | N = 100 | -- | -- | -- |
|  |  | *pgrn-1* | 1.00 ± 0.06 | N = 100 | n.s. | -- | -- |
|  |  | *ced-3(n717)* | 1.00 ± 0.01 | N = 100 | n.s. | n.s. | -- |
|  |  | *ced-9(n1950)* | 1.00 ± 0.03 | N = 100 | n.s. | n.s. | n.s. |
|  |  | *pgrn-1; ced-9* | 1.00 ± 0.04 | N = 100 | n.s. | n.s. | n.s. |
|  |  | *ced-9; ced-3* | 1.00 ± 0.00 | N = 100 | n.s. | n.s. | n.s. |
|  | 2.5 | Control | 0.53 ± 0.07 | N = 100 | -- | -- | -- |
|  |  | *pgrn-1* | 0.91 ± 0.11 | N = 100 | P < 0.001 | -- | -- |
|  |  | *ced-3(n717)* | 0.81 ± 0.10 | N = 100 | P < 0.001 | n.s. | -- |
|  |  | *ced-9(n1950)* | 1.04 ± 0.13 | N = 100 | P < 0.001 | n.s. | P < 0.01 |
|  |  | *pgrn-1; ced-9* | 0.70 ± 0.06 | N = 100 | P < 0.001 | n.s. | P < 0.01 |
|  |  | *ced-9; ced-3* | 0.90 ± 0.06 | N = 100 | P < 0.001 | n.s. | n.s. |
|  | 5 | Control | 0.27 ± 0.13 | N = 100 | -- | **--** | -- |
|  |  | *pgrn-1* | 0.79 ± 0.00 | N = 100 | P < 0.001 | **--** | -- |
|  |  | *ced-3(n717)* | 0.61 ± 0.01 | N = 100 | P < 0.001 | P < 0.05 | -- |
|  |  | *ced-9(n1950)* | 0.68 ± 0.04 | N = 100 | P < 0.001 | n.s. | n.s. |
|  |  | *pgrn-1; ced-9* | 0.48 ± 0.04 | N = 100 | P < 0.001 | n.s. | n.s. |
|  |  | *ced-9; ced-3* | 0.56 ± 0.03 | N = 100 | P < 0.001 | P < 0.01 | n.s. |

| **Table S2D. ER stress resistance of *ced-4(n1162lf)* and *ced-4(n1162lf)* *ced-9(n2812lf)* mutants** | | | | | | | |
| --- | --- | --- | --- | --- | --- | --- | --- |
| **Repeat #** | **Tunicamycin**  **(µg/mL)** | **Genotype** | **Fraction developing to L4 ± SD** | **N** | **P vs.**  **control** | **P vs.  *pgrn-1*** | **P vs. *ced-4*** |
| 1 | 0 | Control | 1.00 ± 0.00 | N = 100 | -- | -- | -- |
|  |  | *pgrn-1* | 1.00 ± 0.00 | N = 100 | n.s. | -- | -- |
|  |  | *ced-4* | 1.00 ± 0.04 | N = 150 | n.s. | n.s. | -- |
|  |  | *ced-4 ced-9* | 1.00 ± 0.01 | N = 100 | n.s. | n.s. | n.s. |
|  | 2.5 | Control | 0.03 ± 0.01 | N = 100 | -- | -- | -- |
|  |  | *pgrn-1* | 0.31 ± 0.04 | N = 100 | P < 0.001 | -- | -- |
|  |  | *ced-4* | 0.33 ± 0.04 | N = 150 | P < 0.001 | n.s. | **--** |
|  |  | *ced-4 ced-9* | 0.29 ± 0.09 | N = 100 | P < 0.001 | n.s. | n.s. |
|  | 5 | Control | 0.00 ± 0.00 | N = 100 | -- | -- | -- |
|  |  | *pgrn-1* | 0.48 ± 0.01 | N = 100 | P < 0.001 | -- | -- |
|  |  | *ced-4* | 0.00 ± 0.00 | N = 150 | P < 0.05 | P < 0.001 | -- |
|  |  | *ced-4 ced-9* | 0.04 ± 0.01 | N = 100 | P < 0.05 | P < 0.001 | n.s. |
| 2 | 0 | Control | 1.00 ± 0.00 | N = 100 | -- | -- | -- |
|  |  | *pgrn-1* | 1.00 ± 0.06 | N = 100 | n.s. | -- | -- |
|  |  | *ced-4 ced-9* | 1.00 ± 0.00 | N = 100 | n.s. | n.s. | -- |
|  | 2.5 | Control | 0.53 ± 0.07 | N = 100 | -- | -- | -- |
|  |  | *pgrn-1* | 0.91 ± 0.11 | N = 100 | P < 0.001 | -- | -- |
|  |  | *ced-4 ced-9* | 0.55 ± 0.03 | N = 100 | n.s. | P < 0.001 | -- |
|  | 5 | Control | 0.27 ± 0.13 | N = 100 | -- | **--** | -- |
|  |  | *pgrn-1* | 0.79 ± 0.00 | N = 100 | P < 0.001 | **--** | -- |
|  |  | *ced-4 ced-9* | 0.34 ± 0.01 | N = 100 | n.s. | P < 0.001 | -- |
| 3* | 0 | Control | 1.00 ± 0.012 | N = 150 | -- | **--** | -- |
|  |  | *pgrn-1* | 1.00 ± 0.035 | N = 150 | n.s. | -- | -- |
|  |  | *ced-4* | 1.00 ± 0.042 | N = 150 | n.s. | n.s. | -- |
|  |  | *ced-4 ced-9* | 1.00 ± 0.031 | N = 150 | n.s. | n.s. | n.s. |
|  | 2 | Control | 0.24 ± 0.031 | N = 150 | -- | **--** | -- |
|  |  | *pgrn-1* | 0.87 ± 0.081 | N = 150 | P < 0.001 | -- | **--** |
|  |  | *ced-4* | 0.11 ± 0.012 | N = 150 | n.s. | P < 0.001 | -- |
|  |  | *ced-4 ced-9* | 0.71 ± 0.060 | N = 150 | P < 0.001 | P < 0.05 | P < 0.001 |
|  | 5 | Control | 0.05 ± 0.042 | N = 150 | -- | **--** | -- |
|  |  | *pgrn-1* | 0.72 ± 0.058 | N = 150 | P < 0.001 | -- | **--** |
|  |  | *ced-4* | 0.00 ± 0.00 | N = 150 | n.s. | P < 0.001 | -- |
|  |  | *ced-4 ced-9* | 0.23 ± 0.023 | N = 150 | P < 0.05 | P < 0.001 | P < 0.01 |
