# Supplementary material for: A Shift to Organismal Stress Resistance in Programmed Cell Death Mutants
Source: PLoS Genet. 2013 Sep 19;9(9):e1003714. doi: 10.1371/journal.pgen.1003714 (PMC3778000; doi:10.1371/journal.pgen.1003714)
Supplement: Table S3 — Related to Figure 3. Engulfment mutants are resistant to unfolded protein stress. (A) Newly laid wild-type control, pgrn-1(tm985), ced-1(e1735), ced-6(n1813) and ced-7(n1892) embryos were collected and placed onto plates with varying doses of tunicamycin. Three days later, the number of animals that had developed to L4 stage was determined. The fraction of animals that developed to L4 stage ± SD are shown. (B) Newly laid wild-type control, pgrn-1(tm985), ced-2(e1752), ced-5(n1812) and ced-10(n3246) embryos were collected and placed onto plates with varying doses of tunicamycin. Three days later, the number of animals that had developed to L4 stage was determined. The fraction of animals that developed to L4 stage ± SD are shown. (C) Day 1 adult wild-type control, ced-1(e1735), ced-6(n1813), ced-7(n1892), ced-2(e1752) and ced-5(n1812) mutants were exposed to osmotic stress with 600 mM NaCl for 24 hours or thermal stress at 35°C for 10 hours and then scored for survival. Shown are mean survival ± SD and p value versus control (ANOVA with Tukey post-test). (D) Newly laid wild-type control, pgrn-1(tm985), abl-1(ok171), abi-1(ok640) and abi-1(tm494) embryos were collected and placed onto plates with varying doses of tunicamycin. Three days later, the number of animals that had developed to L4 stage was determined. The fraction of animals that developed to L4 stage ± SD are shown. P value versus control and pgrn-1 mutant are shown (ANOVA with Bonferroni post-tests). (E) Newly laid wild-type control, pgrn-1(tm985), ced-1(n2091), ced-1(n2091); abi-1(n1963), ced-6(n2095), and ced-6(n2095); abi-1(n1963) embryos were collected and placed onto plates with varying doses of tunicamycin. Three days later, the number of animals that had developed to L4 stage was determined. The fraction of animals that developed to L4 stage ± SD are shown. P value versus control and ced-1 or ced-6 mutants are shown (ANOVA with Bonferroni post-tests). (F) Newly laid wild-type control, pgrn-1(tm985), [file pgen.1003714.s015.docx]

**Supplemental Table S3** *Indicates experiment shown in Figures.

| **Table S3A. ER stress resistance of *ced-1(e1735), ced-6(n1813), ced-7(n1892)*** | | | | | | | |
| --- | --- | --- | --- | --- | --- | --- | --- |
| **Gene** | **Repeat #** | **Tunicamycin**  **(µg/mL)** | **Genotype** | **Fraction developing to L4 ± SD** | **N** | **P vs.**  **control** | **P vs. *pgrn-1*** |
| *ced-1,6,7* | 1* | 0 | Control | 1.00 ± 0.02 | N = 150 | -- | -- |
|  |  |  | *pgrn-1* | 1.00 ± 0.06 | N = 150 | n.s. | -- |
|  |  |  | *ced-1* | 1.00 ± 0.01 | N = 150 | n.s. | n.s. |
|  |  |  | *ced-6* | 1.00 ± 0.03 | N = 150 | n.s. | n.s. |
|  |  |  | *ced-7* | 1.00 ± 0.07 | N = 150 | n.s. | n.s. |
|  |  |  | *pgrn-1 ced-1* | 1.00 ± 0.06 | N = 150 | n.s. | n.s. |
|  |  |  | *pgrn-1; ced-6* | 1.00 ± 0.03 | N = 150 | n.s. | n.s. |
|  |  |  | *pgrn-1; ced-7* | 1.00 ± 0.02 | N = 150 | n.s. | n.s. |
|  |  | 1 | Control | 0.16 ± 0.16 | N = 150 | -- | -- |
|  |  |  | *pgrn-1* | 0.76 ± 0.05 | N = 150 | P < 0.001 | -- |
|  |  |  | *ced-1* | 0.73 ± 0.13 | N = 150 | P < 0.001 | n.s. |
|  |  |  | *ced-6* | 0.58 ± 0.08 | N = 150 | P < 0.001 | n.s. |
|  |  |  | *ced-7* | 0.53 ± 0.02 | N = 150 | P < 0.001 | P < 0.01 |
|  |  |  | *pgrn-1 ced-1* | 0.85 ± 0.12 | N = 150 | P < 0.001 | n.s. |
|  |  |  | *pgrn-1; ced-6* | 0.69 ± 0.12 | N = 150 | P < 0.001 | n.s. |
|  |  |  | *pgrn-1; ced-7* | 0.77 ± 0.06 | N = 150 | P < 0.001 | n.s. |
|  |  | 5 | Control | 0.03 ± 0.02 | N = 150 | -- | -- |
|  |  |  | *pgrn-1* | 0.65 ± 0.10 | N = 150 | P < 0.001 | -- |
|  |  |  | *ced-1* | 0.12 ± 0.01 | N = 150 | n.s. | P < 0.001 |
|  |  |  | *ced-6* | 0.19 ± 0.03 | N = 150 | n.s. | P < 0.001 |
|  |  |  | *ced-7* | 0.24 ± 0.03 | N = 150 | P < 0.01 | P < 0.001 |
|  |  |  | *pgrn-1 ced-1* | 0.47 ± 0.18 | N = 150 | P < 0.001 | n.s. |
|  |  |  | *pgrn-1; ced-6* | 0.33 ± 0.06 | N = 150 | P < 0.001 | P < 0.001 |
|  |  |  | *pgrn-1; ced-7* | 0.36 ± 0.10 | N = 150 | P < 0.001 | P < 0.001 |
| *ced-1* | 2 | 0 | Control | 1.00 ± 0.02 | N = 150 | -- | -- |
|  |  |  | *pgrn-1* | 1.00 ± 0.07 | N = 150 | n.s. | -- |
|  |  |  | *ced-1* | 1.00 ± 0.05 | N = 150 | n.s. | n.s. |
|  |  |  | *pgrn-1 ced-1* | 1.00 ± 0.06 | N = 150 | n.s. | n.s. |
|  |  | 1 | Control | 1.00 ± 0.02 | N = 150 | -- | -- |
|  |  |  | *pgrn-1* | 1.00 ± 0.02 | N = 150 | P < 0.001 | -- |
|  |  |  | *ced-1* | 0.80 ± 0.12 | N = 150 | P < 0.001 | n.s. |
|  |  |  | *pgrn-1 ced-1* | 0.91 ± 0.06 | N = 150 | P < 0.001 | n.s. |
|  |  | 5 | Control | 0.14 ± 0.09 | N = 150 | -- | -- |
|  |  |  | *pgrn-1* | 0.76 ± 0.07 | N = 150 | P < 0.001 | -- |
|  |  |  | *ced-1* | 0.49 ± 0.06 | N = 150 | P < 0.001 | P < 0.001 |
|  |  |  | *pgrn-1 ced-1* | 0.78 ± 0.10 | N = 150 | P < 0.001 | n.s. |
| *ced-6,7* | 2 | 0 | Control | 1.00 ± 0.02 | N = 150 | -- | -- |
|  |  |  | *pgrn-1* | 1.00 ± 0.02 | N = 150 | n.s. | -- |
|  |  |  | *ced-6* | 1.00 ± 0.05 | N = 150 | n.s. | n.s. |
|  |  |  | *ced-7* | 1.00 ± 0.08 | N = 150 | n.s. | n.s. |
|  |  |  | *pgrn-1; ced-6* | 1.00 ± 0.05 | N = 150 | n.s. | n.s. |
|  |  |  | *pgrn-1; ced-7* | 1.00 ± 0.05 | N = 150 | n.s. | n.s. |
|  |  | 1 | Control | 0.14 ± 0.10 | N = 150 | -- | -- |
|  |  |  | *pgrn-1* | 0.93 ± 0.03 | N = 150 | P < 0.001 | **--** |
|  |  |  | *ced-6* | 0.88 ± 0.03 | N = 150 | P < 0.001 | n.s. |
|  |  |  | *ced-7* | 0.75 ± 0.08 | N = 150 | P < 0.001 | P < 0.01 |
|  |  |  | *pgrn-1; ced-6* | 0.85 ± 0.05 | N = 150 | P < 0.001 | n.s. |
|  |  |  | *pgrn-1; ced-7* | 0.99 ± 0.01 | N = 150 | P < 0.001 | n.s. |
|  |  | 5 | Control | 0.11 ± 0.08 | N = 150 | **--** | **--** |
|  |  |  | *pgrn-1* | 0.45 ± 0.15 | N = 150 | P < 0.001 | **--** |
|  |  |  | *ced-6* | 0.17 ± 0.06 | N = 150 | n.s. | P < 0.001 |
|  |  |  | *ced-7* | 0.16 ± 0.02 | N = 150 | n.s. | P < 0.001 |
|  |  |  | *pgrn-1; ced-6* | 0.14 ± 0.06 | N = 150 | n.s. | P < 0.001 |
|  |  |  | *pgrn-1; ced-7* | 0.10 ± 0.04 | N = 150 | n.s. | P < 0.001 |

| **Table S3B. ER stress resistance of *ced-2(1752), ced-5(n1812), ced-10(n3246)*** | | | | | | | |
| --- | --- | --- | --- | --- | --- | --- | --- |
| **Gene** | **Repeat #** | **Tunicamycin**  **(µg/mL)** | **Genotype** | **Fraction developing to L4 ± SD** | **N** | **P vs.**  **control** | **P vs. *pgrn-1*** |
| *ced-2,5,10* | 1* | 0 | Control | 1.00 ± 0.02 | N = 150 | -- | -- |
|  |  |  | *pgrn-1* | 1.00 ± 0.05 | N = 150 | n.s. | -- |
|  |  |  | *ced-2* | 1.00 ± 0.06 | N = 150 | n.s. | n.s. |
|  |  |  | *ced-5* | 1.00 ± 0.12 | N = 150 | n.s. | n.s. |
|  |  |  | *ced-10* | 1.00 ± 0.05 | N = 150 | n.s. | n.s. |
|  |  |  | *pgrn-1; ced-2* | 1.00 ± 0.03 | N = 150 | n.s. | n.s. |
|  |  |  | *pgrn-1; ced-5* | 1.00 ± 0.01 | N = 150 | n.s. | n.s. |
|  |  |  | *pgrn-1; ced-10* | 1.00 ± 0.02 | N = 150 | n.s. | n.s. |
|  |  | 1 | Control | 0.11 ± 0.04 | N = 150 | -- | -- |
|  |  |  | *pgrn-1* | 0.93 ± 0.05 | N = 150 | P < 0.001 | **--** |
|  |  |  | *ced-2* | 0.29 ± 0.09 | N = 150 | P < 0.01 | P < 0.001 |
|  |  |  | *ced-5* | 0.82 ± 0.07 | N = 150 | P < 0.001 | n.s. |
|  |  |  | *ced-10* | 0.30 ± 0.04 | N = 150 | P < 0.01 | P < 0.001 |
|  |  |  | *pgrn-1; ced-2* | 0.72 ± 0.09 | N = 150 | P < 0.001 | P < 0.001 |
|  |  |  | *pgrn-1; ced-5* | 0.76 ± 0.09 | N = 150 | P < 0.001 | P < 0.01 |
|  |  |  | *pgrn-1; ced-10* | 0.60 ± 0.06 | N = 150 | P < 0.001 | P < 0.001 |
|  |  | 5 | Control | 0.04 ± 0.01 | N = 150 | -- | -- |
|  |  |  | *pgrn-1* | 0.63 ± 0.13 | N = 150 | P < 0.001 | -- |
|  |  |  | *ced-2* | 0.04 ± 0.00 | N = 150 | n.s. | P < 0.001 |
|  |  |  | *ced-5* | 0.40 ± 0.02 | N = 150 | P < 0.001 | P < 0.001 |
|  |  |  | *ced-10* | 0.23 ± 0.05 | N = 150 | P < 0.01 | P < 0.001 |
|  |  |  | *pgrn-1; ced-2* | 0.72 ± 0.03 | N = 150 | P < 0.001 | n.s. |
|  |  |  | *pgrn-1; ced-5* | 0.49 ± 0.05 | N = 150 | P < 0.001 | n.s. |
|  |  |  | *pgrn-1; ced-10* | 0.36 ± 0.05 | N = 150 | P < 0.001 | P < 0.001 |
| *ced-5* | 2 | 0 | Control | 1.00 ± 0.01 | N = 150 | -- | -- |
|  |  |  | *pgrn-1* | 1.00 ± 0.03 | N = 150 | n.s. | -- |
|  |  |  | *ced-5* | 1.00 ± 0.01 | N = 150 | n.s. | n.s. |
|  |  |  | *pgrn-1; ced-5* | 1.00 ± 0.07 | N = 150 | n.s. | n.s. |
|  |  | 1 | Control | 0.26 ± 0.07 | N = 150 | -- | -- |
|  |  |  | *pgrn-1* | 0.96 ± 0.02 | N = 150 | P < 0.001 | -- |
|  |  |  | *ced-5* | 0.81 ± 0.05 | N = 150 | P < 0.001 | P < 0.001 |
|  |  |  | *pgrn-1; ced-5* | 0.87 ± 0.06 | N = 150 | P < 0.001 | n.s. |
|  |  | 5 | Control | 0.13 ± 0.04 | N = 150 | -- | -- |
|  |  |  | *pgrn-1* | 0.57 ± 0.08 | N = 150 | P < 0.001 | -- |
|  |  |  | *ced-5* | 0.33 ± 0.06 | N = 150 | P < 0.001 | P < 0.001 |
|  |  |  | *pgrn-1; ced-5* | 0.37 ± 0.04 | N = 150 | P < 0.001 | P < 0.001 |
| *ced-2,10* | 2 | 0 | Control | 1.00 ± 0.03 | N = 150 | -- | -- |
|  |  |  | *pgrn-1* | 1.00 ± 0.05 | N = 150 | n.s. | -- |
|  |  |  | *ced-2* | 1.00 ± 0.03 | N = 150 | n.s. | n.s. |
|  |  |  | *ced-10* | 1.00 ± 0.03 | N = 150 | n.s. | n.s. |
|  |  |  | *pgrn-1; ced-2* | 1.00 ± 0.05 | N = 150 | n.s. | n.s. |
|  |  |  | *pgrn-1; ced-10* | 1.00 ± 0.05 | N = 150 | n.s. | n.s. |
|  |  | 1 | Control | 0.07 ± 0.01 | N = 150 | -- | -- |
|  |  |  | *pgrn-1* | 0.84 ± 0.04 | N = 150 | P < 0.001 | -- |
|  |  |  | *ced-2* | 0.64 ± 0.01 | N = 150 | P < 0.001 | P < 0.001 |
|  |  |  | *ced-10* | 0.35 ± 0.04 | N = 150 | P < 0.001 | P < 0.001 |
|  |  |  | *pgrn-1; ced-2* | 1.02 ± 0.02 | N = 150 | P < 0.001 | P < 0.001 |
|  |  |  | *pgrn-1; ced-10* | 0.50 ± 0.01 | N = 150 | P < 0.001 | P < 0.001 |
|  |  | 5 | Control | 0.04 ± 0.00 | N = 150 | -- | -- |
|  |  |  | *pgrn-1* | 0.89 ± 0.04 | N = 150 | P < 0.001 | -- |
|  |  |  | *ced-2* | 0.17 ± 0.03 | N = 150 | P < 0.01 | P < 0.001 |
|  |  |  | *ced-10* | 0.15 ± 0.03 | N = 150 | P < 0.01 | P < 0.001 |
|  |  |  | *pgrn-1; ced-2* | 0.78 ± 0.08 | N = 150 | P < 0.001 | P < 0.01 |
|  |  |  | *pgrn-1; ced-10* | 0.21 ± 0.05 | N = 150 | P < 0.01 | P < 0.001 |
| *ced-2,10* | 3 | 0 | Control | 1.00 ± 0.05 | N = 150 | -- | -- |
|  |  |  | *pgrn-1* | 1.00 ± 0.00 | N = 150 | n.s. | -- |
|  |  |  | *ced-2* | 1.00 ± 0.05 | N = 150 | n.s. | n.s. |
|  |  |  | *ced-10* | 1.00 ± 0.12 | N = 150 | n.s. | n.s. |
|  |  |  | *pgrn-1; ced-2* | 1.00 ± 0.09 | N = 150 | n.s. | n.s. |
|  |  |  | *pgrn-1; ced-10* | 1.00 ± 0.01 | N = 150 | n.s. | n.s. |
|  |  | 1 | Control | 0.11 ± 0.04 | N = 150 | -- | -- |
|  |  |  | *pgrn-1* | 0.90 ± 0.12 | N = 150 | P < 0.001 | **--** |
|  |  |  | *ced-2* | 0.57 ± 0.15 | N = 150 | P < 0.001 | P < 0.001 |
|  |  |  | *ced-10* | 0.90 ± 0.05 | N = 150 | P < 0.001 | n.s. |
|  |  |  | *pgrn-1; ced-2* | 0.92 ± 0.05 | N = 150 | P < 0.001 | n.s. |
|  |  |  | *pgrn-1; ced-10* | 0.78 ± 0.06 | N = 150 | P < 0.001 | n.s. |
|  |  | 5 | Control | 0.03 ± 0.02 | N = 150 | **--** | **--** |
|  |  |  | *pgrn-1* | 0.60 ± 0.20 | N = 150 | P < 0.001 | **--** |
|  |  |  | *ced-2* | 0.07 ± 0.01 | N = 150 | n.s. | P < 0.001 |
|  |  |  | *ced-10* | 0.65 ± 0.22 | N = 150 | P < 0.001 | n.s. |
|  |  |  | *pgrn-1; ced-2* | 0.68 ± 0.10 | N = 150 | P < 0.001 | n.s. |
|  |  |  | *pgrn-1; ced-10* | 0.65 ± 0.05 | N = 150 | P < 0.001 | n.s. |
|  |  |  |  |  |  |  |  |

| **Table S3C. Effect of engulfment mutation on heat and osmotic stress resistance** | | | | | |
| --- | --- | --- | --- | --- | --- |
| **Treatment** | **Repeat #** | **Genotype** | **Mean survival ± SD** | **N** | **P vs.**  **control** |
| Osmotic Stress | 1* | Control | 0.29 ± 0.10 | N = 80 | -- |
|  |  | *ced-1* | 0.50 ± 0.07 | N = 80 | P < 0.05 |
|  |  | *ced-2* | 0.70 ± 0.10 | N = 80 | P < 0.0001 |
|  | 2 | Control | 0.39 ± 0.01 | N = 60 | -- |
|  |  | *ced-1* | 0.67 ± 0.18 | N = 60 | n.s. |
|  |  | *ced-2* | 0.59 ± 0.11 | N = 60 | n.s. |
|  | 3 | Control | 0.30 ± 0.001 | N = 40 | -- |
|  |  | *ced-1* | 0.55 ± 0.14 | N = 40 | P = 0.13 |
|  |  | *ced-6* | 0.35 ± 0.28 | N = 40 | n.s. |
|  |  | *ced-7* | 0.68 ± 0.11 | N = 40 | P < 0.05 |
|  |  | *ced-2* | 0.58 ± 0.04 | N = 40 | P < 0.01 |
|  |  | *ced-5* | 0.30 ± 0.14 | N = 40 | n.s. |
|  |  |  |  |  |  |
| Thermotolerance | 1* | Control | 0.18 ± 0.17 | N = 40 | -- |
|  |  | *ced-1* | 0.28 ± 0.18 | N = 40 | n.s. |
|  |  | *ced-2* | 0.90 ± 0.07 | N = 40 | P = 0.03 |
|  | 2 | Control | 0.16 ± 0.05 | N = 80 | -- |
|  |  | *ced-1* | 0.30 ± 0.30 | N = 80 | n.s. |
|  | 3 | Control | 0.27 ± 0.24 | N = 60 | -- |
|  |  | *ced-2* | 0.01 ± 0.01 | N = 60 | n.s. |

| **Table S3D. ER stress resistance of *abl-1(ok171), abi-1(ok640)* and *abi-1(tm494)*** | | | | | | | |
| --- | --- | --- | --- | --- | --- | --- | --- |
| **Treatment** | **Repeat #** | **Tunicamycin**  **(µg/mL)** | **Genotype** | **Fraction developing to L4 ± SD** | **N** | **P vs.**  **control** | **P vs. *pgrn-1*** |
| ER stress | 1* | 0 | Control | 1.00 ± 0.02 | N = 150 | -- | -- |
|  |  |  | *pgrn-1* | 1.00 ± 0.07 | N = 150 | n.s. | -- |
|  |  |  | *abl-1* | 1.00 ± 0.06 | N = 150 | n.s. | n.s. |
|  |  |  | *abi-1(ok640)* | 1.00 ± 0.02 | N = 150 | n.s. | n.s. |
|  |  |  | *abi-1(tm494)* | 1.00 ± 0.04 | N = 150 | n.s. | n.s. |
|  |  | 1 | Control | 0.13 ± 0.04 | N = 150 | -- | -- |
|  |  |  | *pgrn-1* | 0.84 ± 0.05 | N = 150 | P < 0.001 | -- |
|  |  |  | *abl-1* | 0.77 ± 0.13 | N = 150 | P < 0.001 | n.s. |
|  |  |  | *abi-1(ok640)* | 0.73 ± 0.11 | N = 150 | P < 0.001 | n.s. |
|  |  |  | *abi-1(tm494)* | 0.55 ± 0.16 | N = 150 | P < 0.001 | P < 0.01 |
|  |  | 5 | Control | 0.14 ± 0.09 | N = 150 | -- | -- |
|  |  |  | *pgrn-1* | 0.76 ± 0.07 | N = 150 | P < 0.001 | -- |
|  |  |  | *abl-1* | 0.38 ± 0.14 | N = 150 | P < 0.01 | P < 0.001 |
|  |  |  | *abi-1(ok640)* | 0.23 ± 0.05 | N = 150 | n.s. | P < 0.001 |
|  |  |  | *abi-1(tm494)* | 0.33 ± 0.11 | N = 150 | P < 0.05 | P < 0.001 |

| **Table S3E. Stress resistance correlates with engulfment defect** | | | | | | | |
| --- | --- | --- | --- | --- | --- | --- | --- |
| **Treatment** | **Repeat #** | **Tunicamycin**  **(µg/mL)** | **Genotype** | **Fraction developing to L4 ± SD** | **N** | **P vs.**  **control** | **P vs. *ced-1 or ced-6*** |
| ER stress | 1* | 0 | Control | 1.00 ± 0.046 | N = 150 | -- | -- |
|  |  |  | *pgrn-1* | 1.00 ± 0.087 | N = 150 | n.s. | -- |
|  |  |  | *ced-1(n2091)* | 1.00 ± 0.072 | N = 150 | n.s. | -- |
|  |  |  | *ced-1(n2091); abl-1(n1963)* | 1.00 ± 0.020 | N = 150 | n.s. | n.s. |
|  |  |  | *ced-6(n2095)* | 0.52 ± 0.031 | N = 150 | n.s. | -- |
|  |  |  | *ced-6(n2095); abl-1(ok171)* | 0.82 ± 0.040 | N = 150 | n.s. | n.s. |
|  |  | 2 | Control | 0.59 ± 0.122 | N = 150 | -- | -- |
|  |  |  | *pgrn-1* | 0.88 ± 0.069 | N = 150 | P < 0.001 | -- |
|  |  |  | *ced-1(n2091)* | 0.73 ± 0.111 | N = 150 | n.s. | -- |
|  |  |  | *ced-1(n2091); abl-1(n1963)* | 0.57 ± 0.061 | N = 150 | n.s. | P < 0.05 |
|  |  |  | *ced-6(n2095)* | 0.44 ± 0.092 | N = 150 | P < 0.05 | -- |
|  |  |  | *ced-6(n2095); abl-1(ok171)* | 0.26 ± 0.020 | N = 150 | P < 0.001 | P < 0.05 |
|  |  | 5 | Control | 0.22 ± 0.023 | N = 150 | -- | -- |
|  |  |  | *pgrn-1* | 0.54 ± 0.142 | N = 150 | P < 0.001 | -- |
|  |  |  | *ced-1(n2091)* | 0.10 ± 0.050 | N = 150 | n.s. | -- |
|  |  |  | *ced-1(n2091); abl-1(n1963)* | 0.07 ± 0.035 | N = 150 | P < 0.05 | n.s. |
|  |  |  | *ced-6(n2095)* | 0.16 ± 0.050 | N = 150 | n.s. | -- |
|  |  |  | *ced-6(n2095); abl-1(ok171)* | 0.01 ± 0.012 | N = 150 | P < 0.01 | P < 0.05 |
|  | 2 | 0 | Control | 1.00 ± 0.040 | N = 150 | -- | -- |
|  |  |  | *ced-1(n2091)* | 1.00 ± 0.061 | N = 150 | n.s. | -- |
|  |  |  | *ced-1(n2091); abl-1(n1963)* | 1.00 ± 0.076 | N = 150 | n.s. | n.s. |
|  |  |  | *ced-6(n2095)* | 1.00 ± 0.090 | N = 150 | n.s. | -- |
|  |  |  | *ced-6(n2095); abl-1(ok171)* | 1.00 ± 0.023 | N = 150 | n.s. | -- |
|  |  | 2 | Control | 0.71 ± 0.040 | N = 150 | -- | -- |
|  |  |  | *ced-1(n2091)* | 0.63 ± 0.050 | N = 150 | n.s. | -- |
|  |  |  | *ced-1(n2091); abl-1(n1963)* | 0.48 ± 0.070 | N = 150 | P < 0.001 | P < 0.01 |
|  |  |  | *ced-6(n2095)* | 0.62 ± 0.020 | N = 150 | n.s. | -- |
|  |  |  | *ced-6(n2095); abl-1(ok171)* | 0.34 ± 0.012 | N = 150 | P < 0.001 | P < 0.001 |
|  |  | 5 | Control | 0.19 ± 0.020 | N = 150 | -- | -- |
|  |  |  | *ced-1(n2091)* | 0.11 ± 0.042 | N = 150 | n.s. | -- |
|  |  |  | *ced-1(n2091); abl-1(n1963)* | 0.03 ± 0.031 | N = 150 | P < 0.01 | n.s. |
|  |  |  | *ced-6(n2095)* | 0.02 ± 0.020 | N = 150 | P < 0.01 | -- |
|  |  |  | *ced-6(n2095); abl-1(ok171)* | 0.00 ± 0.000 | N = 150 | P < 0.001 | n.s. |

| **Table S3F. Engulfment genes not required for ER stress resistance** | | | | | | | |
| --- | --- | --- | --- | --- | --- | --- | --- |
| **Treatment** | **Repeat #** | **Tunicamycin**  **(µg/mL)** | **Genotype** | **Fraction developing to L4 ± SD** | **N** | **P vs.**  **control** | **P vs. *pgrn-1*** |
| ER stress | 1 | 0 | Control | 1.00 ± 0.012 | N = 150 | -- | -- |
|  |  |  | *pgrn-1* | 1.00 ± 0.042 | N = 150 | n.s. | -- |
|  |  |  | *unc-53* | 1.00 ± 0.012 | N = 75 | n.s. | n.s. |
|  |  |  | *unc-73* | 1.00 ± 0.042 | N = 150 | n.s. | n.s. |
|  |  | 2 | Control | 0.52 ± 0.216 | N = 150 | -- | -- |
|  |  |  | *pgrn-1* | 0.82 ± 0.110 | N = 150 | P < 0.01 | -- |
|  |  |  | *unc-53* | 0.54 ± 0.061 | N = 75 | n.s. | P < 0.01 |
|  |  |  | *unc-73* | 0.59 ± 0.101 | N = 150 | n.s. | P < 0.05 |
|  | 2 | 0 | Control | 1.00 ± 0.092 | N = 150 | -- | -- |
|  |  |  | *pgrn-1* | 1.00 ± 0.042 | N = 150 | n.s. | -- |
|  |  |  | *unc-53* | 1.00 ± 0.167 | N = 75 | n.s. |  |
|  |  |  | *unc-73* | 1.00 ± 0.100 | N = 150 | n.s. |  |
|  |  | 2 | Control | 0.62 ± 0.072 | N = 150 | -- | -- |
|  |  |  | *pgrn-1* | 0.62 ± 0.092 | N = 150 | n.s. | -- |
|  |  |  | *unc-53* | 0.25 ± 0.139 | N = 75 | P < 0.001 | P < 0.001 |
|  |  |  | *unc-73* | 0.26 ± 0.069 | N = 150 | P < 0.001 | P < 0.001 |
|  | 3* | 0 | Control | 1.00 ± 0.046 | N = 150 | -- | -- |
|  |  |  | *pgrn-1* | 1.00 ± 0.087 | N = 150 | n.s. | -- |
|  |  |  | *unc-53* | 1.00 ± 0.083 | N = 75 | n.s. |  |
|  |  |  | *unc-73* | 1.00 ± 0.130 | N = 150 | n.s. |  |
|  |  | 2 | Control | 0.59 ± 0.122 | N = 150 | -- | -- |
|  |  |  | *pgrn-1* | 0.88 ± 0.069 | N = 150 | P < 0.01 | -- |
|  |  |  | *unc-53* | 0.72 ± 0.080 | N = 75 | n.s. | n.s. |
|  |  |  | *unc-73* | 0.66 ± 0.103 | N = 150 | n.s. | P < 0.05 |
|  |  | 5 | Control | 0.22 ± 0.023 | N = 150 | -- | -- |
|  |  |  | *pgrn-1* | 0.54 ± 0.142 | N = 150 | P < 0.001 | -- |
|  |  |  | *unc-53* | 0.16 ± 0.069 | N = 75 | n.s. | P < 0.001 |
|  |  |  | *unc-73* | 0.40 ± 0.000 | N = 150 | n.s. | n.s. |

| **Table S3G. *pqn-41* mutations confer ER stress resistance at low doses of Tunicamycin** | | | | | | | |
| --- | --- | --- | --- | --- | --- | --- | --- |
| **Treatment** | **Repeat #** | **Tunicamycin**  **(µg/mL)** | **Genotype** | **Fraction developing to L4 ± SD** | **N** | **P vs.**  **control** | **P vs. *pgrn-1*** |
| ER stress | 1* | 0 | Control | 1.00 ± 0.012 | N = 150 | -- | -- |
|  |  |  | *pgrn-1* | 1.00 ± 0.035 | N = 150 | n.s. | -- |
|  |  |  | *pqn-41* | 1.00 ± 0.081 | N = 150 | n.s. | n.s. |
|  |  |  | *pgrn-1; pqn-41* | 1.00 ± 0.099 | N = 150 | n.s. | n.s. |
|  |  | 2 | Control | 0.24 ± 0.031 | N = 150 | -- | -- |
|  |  |  | *pgrn-1* | 0.87 ± 0.081 | N = 150 | P < 0.001 | -- |
|  |  |  | *pqn-41* | 0.42 ± 0.129 | N = 150 | P < 0.05 | P < 0.001 |
|  |  |  | *pgrn-1; pqn-41* | 0.94 ± 0.120 | N = 150 | P < 0.001 | n.s. |
|  |  | 5 | Control | 0.05 ± 0.042 | N = 150 | -- | -- |
|  |  |  | *pgrn-1* | 0.72 ± 0.058 | N = 150 | P < 0.001 | -- |
|  |  |  | *pqn-41* | 0.12 ± 0.050 | N = 150 | n.s. | P < 0.001 |
|  |  |  | *pgrn-1; pqn-41* | 0.31 ± 0.151 | N = 150 | P < 0.001 | P < 0.001 |
