# Supplementary material for: A Shift to Organismal Stress Resistance in Programmed Cell Death Mutants
Source: PLoS Genet. 2013 Sep 19;9(9):e1003714. doi: 10.1371/journal.pgen.1003714 (PMC3778000; doi:10.1371/journal.pgen.1003714)
Supplement: Table S4 — Related to Figure 4. pgrn-1(-) resistance to ER stress may be partially dependent on the UPR pathway, daf-16 and pmk-1. (A) Newly laid embryos from wild-type control, pgrn-1(tm985), ire-1(ok799) and pgrn-1(-); ire-1(-) mutants were collected and placed onto plates with varying doses of tunicamycin. Three days later, the number of animals that had developed to L4 stage was determined. The fraction of animals that developed to L4 stage ± SD is shown. P value versus control and pgrn-1 mutant are shown (ANOVA with Bonferroni post-tests). (B) Newly laid embryos from wild-type control, pgrn-1(tm985), daf-2(e1370), pgrn-1(-);daf-2(-), daf-16(mu86) and pgrn-1(-); daf-16(-) mutants were collected and placed onto plates with varying doses of tunicamycin. Three days later, the number of animals that had developed to L4 stage was determined. The fraction of animals that developed to L4 stage ± SD is shown. P value versus control and pgrn-1 mutant are shown (ANOVA with Bonferroni post-tests). (C) Newly laid embryos from wild-type control, pgrn-1(tm985), daf-16(mu86); muIs113 and pgrn-1(tm985); muIs113 mutants were collected and placed onto plates with varying doses of tunicamycin. Three days later, the number of animals that had developed to L4 stage was determined. The fraction of animals that developed to L4 stage ± SD is shown. P value versus control and pgrn-1 mutant are shown (ANOVA with Bonferroni post-tests). (D) Newly laid embryos from wild-type control, pgrn-1(tm985), pmk-1(km25) and pgrn-1(-); pmk-1(-) mutants were collected and placed onto plates with varying doses of tunicamycin. Three days later, the number of animals that had developed to L4 stage was determined. The fraction of animals that developed to L4 stage ± SD is shown. P value versus control and pgrn-1 mutant are shown (ANOVA with Bonferroni post-tests). (DOCX) [file pgen.1003714.s016.docx]

**Supplemental Table S4** *Indicates experiment shown in Figures.

| **Table S4A. Genes required for *pgrn-1(-)* resistance to ER stress** | | | | | | | |
| --- | --- | --- | --- | --- | --- | --- | --- |
| **Treatment** | **Repeat #** | **Tunicamycin**  **(µg/mL)** | **Genotype** | **Fraction developing to L4 ± SD** | **N** | **P vs.**  **control** | **P vs. *pgrn-1*** |
| ER stress | 1* | 0 | Control | 1.00 ± 0.02 | N = 150 | -- | -- |
|  |  |  | *pgrn-1* | 1.00 ± 0.01 | N = 150 | n.s. | -- |
|  |  |  | *ire-1* | 1.00 ± 0.09 | N = 150 | n.s. | n.s. |
|  |  |  | *pgrn-1; ire-1* | 1.00 ± 0.02 | N = 150 | n.s. | n.s. |
|  |  | 1 | Control | 0.09 ± 0.07 | N = 150 | -- | -- |
|  |  |  | *pgrn-1* | 0.62 ± 0.02 | N = 150 | P < 0.001 | -- |
|  |  |  | *ire-1* | 0.07 ± 0.02 | N = 150 | n.s. | P < 0.001 |
|  |  |  | *pgrn-1; ire-1* | 0.01 ± 0.02 | N = 150 | n.s. | P < 0.001 |
|  |  | 5 | Control | 0.07 ± 0.05 | N = 150 | -- | -- |
|  |  |  | *pgrn-1* | 0.68 ± 0.05 | N = 150 | P < 0.001 | -- |
|  |  |  | *ire-1* | 0.00 ± 0.00 | N = 150 | n.s. | P < 0.001 |
|  |  |  | *pgrn-1; ire-1* | 0.00 ± 0.00 | N = 150 | n.s. | P < 0.001 |

| **Table S4B. Genes required for *pgrn-1(-)* resistance to ER stress** | | | | | | | |
| --- | --- | --- | --- | --- | --- | --- | --- |
| **Treatment** | **Repeat #** | **Tunicamycin**  **(µg/mL)** | **Genotype** | **Fraction developing to L4 ± SD** | **N** | **P vs.**  **control** | **P vs. *pgrn-1*** |
| ER stress | 1* | 0 | Control | 1.00 ± 0.01 | N = 150 | -- | -- |
|  |  |  | *pgrn-1* | 1.00 ± 0.01 | N = 150 | n.s. | -- |
|  |  |  | *daf-2* | 1.00 ± 0.03 | N = 150 | n.s. | n.s. |
|  |  |  | *pgrn-1; daf-2* | 1.00 ± 0.00 | N = 150 | n.s. | n.s. |
|  |  |  | *daf-16* | 1.00 ± 0.04 | N = 150 | n.s. | n.s. |
|  |  |  | *daf-16 pgrn-1* | 1.00 ± 0.02 | N = 150 | n.s. | n.s. |
|  |  | 1 | Control | 0.06 ± 0.02 | N = 150 | -- | -- |
|  |  |  | *pgrn-1* | 0.51 ± 0.07 | N = 150 | P < 0.001 | -- |
|  |  |  | *daf-2* | 0.96 ± 0.00 | N = 150 | P < 0.001 | P < 0.001 |
|  |  |  | *pgrn-1; daf-2* | 0.79 ± 0.10 | N = 150 | P < 0.001 | P < 0.001 |
|  |  |  | *daf-16* | 0.23 ± 0.06 | N = 150 | P < 0.001 | P < 0.001 |
|  |  |  | *daf-16 pgrn-1* | 0.12 ± 0.03 | N = 150 | n.s. | P < 0.001 |
|  |  | 5 | Control | 0.02 ± 0.02 | N = 150 | -- | -- |
|  |  |  | *pgrn-1* | 0.44 ± 0.06 | N = 150 | P < 0.001 | -- |
|  |  |  | *daf-2* | 0.96 ± 0.01 | N = 150 | P < 0.001 | P < 0.001 |
|  |  |  | *pgrn-1; daf-2* | 0.80 ± 0.05 | N = 150 | P < 0.001 | P < 0.001 |
|  |  |  | *daf-16* | 0.12 ± 0.03 | N = 150 | P < 0.05 | P < 0.001 |
|  |  |  | *daf-16 pgrn-1* | 0.07 ± 0.02 | N = 150 | n.s. | P < 0.001 |
|  | 2 | 0 | Control | 1.00 ± 0.042 | N = 150 | -- | -- |
|  |  |  | *pgrn-1* | 1.00 ± 0.076 | N = 150 | n.s. | -- |
|  |  |  | *daf-2* | 1.00 ± 0.083 | N = 150 | n.s. | n.s. |
|  |  |  | *pgrn-1; daf-2* | 1.00 ± 0.064 | N = 150 | n.s. | n.s. |
|  |  | 2 | Control | 0.57 ± 0.110 | N = 150 | -- | -- |
|  |  |  | *pgrn-1* | 0.83 ± 0.061 | N = 150 | P < 0.001 | -- |
|  |  |  | *daf-2* | 0.93 ± 0.122 | N = 150 | P < 0.001 | n.s. |
|  |  |  | *pgrn-1; daf-2* | 1.03 ± 0.072 | N = 150 | P < 0.001 | n.s. |
|  |  | 5 | Control | 0.19 ± 0.000 | N = 150 | -- | -- |
|  |  |  | *pgrn-1* | 0.49 ± 0.122 | N = 150 | P < 0.001 | -- |
|  |  |  | *daf-2* | 0.62 ± 0.200 | N = 150 | P < 0.001 | n.s. |
|  |  |  | *pgrn-1; daf-2* | 0.56 ± 0.095 | N = 150 | P < 0.001 | n.s. |
|  | 3 | 0 | Control | 1.00 ± 0.012 | N = 150 | -- | -- |
|  |  |  | *pgrn-1* | 1.00 ± 0.042 | N = 150 | n.s. | -- |
|  |  |  | *daf-2* | 1.00 ± 0.031 | N = 150 | n.s. | n.s. |
|  |  |  | *pgrn-1; daf-2* | 1.00 ± 0.023 | N = 150 | n.s. | n.s. |
|  |  | 2 | Control | 0.52 ± 0.216 | N = 150 | -- | -- |
|  |  |  | *pgrn-1* | 0.80 ± 0.110 | N = 150 | P < 0.01 | -- |
|  |  |  | *daf-2* | 1.26 ± 0.111 | N = 150 | P < 0.001 | P < 0.001 |
|  |  |  | *pgrn-1; daf-2* | 0.85 ± 0.053 | N = 150 | P < 0.001 | n.s. |
|  | 4 | 0 | Control | 1.00 ± 0.035 | N = 150 | -- | -- |
|  |  |  | *pgrn-1* | 1.00 ± 0.061 | N = 150 | n.s. | -- |
|  |  |  | *daf-2* | 1.00 ± 0.020 | N = 150 | n.s. | n.s. |
|  |  |  | *pgrn-1; daf-2* | 1.00 ± 0.050 | N = 150 | n.s. | n.s. |
|  |  |  | *daf-16* | 1.00 ± 0.058 | N = 150 | n.s. | n.s. |
|  |  |  | *daf-16 pgrn-1* | 1.00 ± 0.053 | N = 150 | n.s. | n.s. |
|  |  | 2 | Control | 0.41 ± 0.130 | N = 150 | -- | -- |
|  |  |  | *pgrn-1* | 0.88 ± 0.069 | N = 150 | P < 0.001 | -- |
|  |  |  | *daf-2* | 0.86 ± 0.070 | N = 150 | P < 0.001 | n.s. |
|  |  |  | *pgrn-1; daf-2* | 1.06 ± 0.042 | N = 150 | P < 0.001 | P < 0.05 |
|  |  |  | *daf-16* | 0.56 ± 0.060 | N = 150 | n.s. | P<0.001 |
|  |  |  | *daf-16 pgrn-1* | 0.43 ± 0.040 | N = 150 | n.s. | P < 0.001 |
|  |  | 5 | Control | 0.40 ± 0.053 | N = 150 | -- | -- |
|  |  |  | *pgrn-1* | 0.82 ± 0.072 | N = 150 | P < 0.001 | -- |
|  |  |  | *daf-2* | 0.81 ± 0.061 | N = 150 | P < 0.001 | n.s. |
|  |  |  | *pgrn-1; daf-2* | 0.64 ± 0.106 | N = 150 | P < 0.01 | P < 0.05 |
|  |  |  | *daf-16* | 0.63 ± 0.150 | N = 150 | P < 0.01 | P<0.05 |
|  |  |  | *daf-16 pgrn-1* | 0.46 ± 0.050 | N = 150 | n.s. | P < 0.001 |

| **Table S4C. Constitutively active DAF-16 does not affect ER stress resistance of *pgrn-1* mutants** | | | | | | | |
| --- | --- | --- | --- | --- | --- | --- | --- |
| **Treatment** | **Repeat #** | **Tunicamycin**  **(µg/mL)** | **Genotype** | **Fraction developing to L4 ± SD** | **N** | **P vs.**  **control** | **P vs. *pgrn-1*** |
| ER stress | 1* | 0 | Control | 1.00 ± 0.042 | N = 150 | -- | -- |
|  |  |  | *pgrn-1* | 1.00 ± 0.076 | N = 150 | n.s. | -- |
|  |  |  | *daf-16; muIs113* | 1.00 ± 0.042 | N = 150 | n.s. | n.s. |
|  |  |  | *pgrn-1; muIs113* | 1.00 ± 0.031 | N = 150 | n.s. | n.s. |
|  |  | 2 | Control | 0.57 ± 0.110 | N = 150 | -- | -- |
|  |  |  | *pgrn-1* | 0.83 ± 0.061 | N = 150 | P < 0.001 | -- |
|  |  |  | *daf-16; muIs113* | 0.56 ± 0.042 | N = 150 | n.s. | P < 0.001 |
|  |  |  | *pgrn-1; muIs113* | 0.76 ± 0.031 | N = 150 | P < 0.05 | n.s. |
|  |  | 5 | Control | 0.19 ± 0.000 | N = 150 | -- | -- |
|  |  |  | *pgrn-1* | 0.49 ± 0.122 | N = 150 | P < 0.001 | -- |
|  |  |  | *daf-16; muIs113* | 0.34 ± 0.064 | N = 150 | n.s. | n.s. |
|  |  |  | *pgrn-1; muIs113* | 0.62 ± 0.053 | N = 150 | P < 0.001 | n.s. |

| **Table S4D. Genes required for *pgrn-1(-)* resistance to ER stress** | | | | | | | |
| --- | --- | --- | --- | --- | --- | --- | --- |
| **Treatment** | **Repeat #** | **Tunicamycin**  **(µg/mL)** | **Genotype** | **Fraction developing to L4 ± SD** | **N** | **P vs.**  **control** | **P vs.**  ***pgrn-1*** |
| ER stress | 1* | 0 | Control | 1.00 ± 0.05 | N = 150 | -- | -- |
|  |  |  | *pgrn-1* | 1.00 ± 0.07 | N = 150 | n.s. | -- |
|  |  |  | *pmk-1* | 1.00 ± 0.02 | N = 150 | n.s. | n.s. |
|  |  |  | *pgrn-1; pmk-1* | 1.00 ± 0.05 | N = 150 | n.s. | n.s. |
|  |  | 1 | Control | 0.14 ± 0.06 | N = 150 | -- | -- |
|  |  |  | *pgrn-1* | 0.67 ± 0.11 | N = 150 | P < 0.001 | -- |
|  |  |  | *pmk-1* | 0.01 ± 0.01 | N = 150 | n.s. | P < 0.001 |
|  |  |  | *pgrn-1; pmk-1* | 0.00 ± 0.00 | N = 150 | n.s. | P < 0.001 |
|  |  | 5 | Control | 0.06 ± 0.02 | N = 150 | -- | -- |
|  |  |  | *pgrn-1* | 0.31 ± 0.12 | N = 150 | P < 0.001 | -- |
|  |  |  | *pmk-1* | 0.00 ± 0.00 | N = 150 | n.s. | P < 0.001 |
|  |  |  | *pgrn-1; pmk-1* | 0.00 ± 0.00 | N = 150 | n.s. | P < 0.001 |
